# Validation of the Recording of Acute Exacerbations of COPD in UK Primary Care Electronic Healthcare Records

**DOI:** 10.1371/journal.pone.0151357

**Published:** 2016-03-09

**Authors:** Kieran J. Rothnie, Hana Müllerová, John R. Hurst, Liam Smeeth, Kourtney Davis, Sara L. Thomas, Jennifer K. Quint

**Affiliations:** 1 Respiratory Epidemiology, Occupational Medicine and Public Health, National Heart and Lung Institute, Imperial College London, London, United Kingdom; 2 Faculty of Epidemiology and Population Health, London School of Hygiene and Tropical Medicine, London, United Kingdom; 3 Respiratory Epidemiology, GSK R&D, Uxbridge, United Kingdom; 4 UCL Respiratory Medicine, University College London, London, United Kingdom; 5 Respiratory Epidemiology, GSK R&D, Collegeville, PA, United States of America; Lee Kong Chian School of Medicine, SINGAPORE

## Abstract

**Background:**

Acute Exacerbations of COPD (AECOPD) identified from electronic healthcare records (EHR) are important for research, public health and to inform healthcare utilisation and service provision. However, there is no standardised method of identifying AECOPD in UK EHR. We aimed to validate the recording of AECOPD in UK EHR.

**Methods:**

We randomly selected 1385 patients with COPD from the Clinical Practice Research Datalink. We selected dates of possible AECOPD based on 15 different algorithms between January 2004 and August 2013. Questionnaires were sent to GPs asking for confirmation of their patients’ AECOPD on the dates identified and for any additional relevant information. Responses were reviewed independently by two respiratory physicians. Positive predictive value (PPV) and sensitivity were calculated.

**Results:**

The response rate was 71.3%. AECOPD diagnostic codes, lower respiratory tract infection (LRTI) codes, and prescriptions of antibiotics and oral corticosteroids (OCS) together for 5–14 days had a high PPV (>75%) for identifying AECOPD. Symptom-based algorithms and prescription of antibiotics or OCS alone had lower PPVs (60–75%). A combined strategy of antibiotic and OCS prescriptions for 5–14 days, or LRTI or AECOPD code resulted in a PPV of 85.5% (95% CI, 82.7–88.3%) and a sensitivity of 62.9% (55.4–70.4%).

**Conclusion:**

Using a combination of diagnostic and therapy codes, the validity of AECOPD identified from EHR can be high. These strategies are useful for understanding health-care utilisation for AECOPD, informing service provision and for researchers. These results highlight the need for common coding strategies to be adopted in primary care to allow easy and accurate identification of events.

## Introduction

Chronic obstructive pulmonary disease (COPD) is a common, progressive disease characterised by airflow obstruction which is not fully reversible. As the third leading cause of death worldwide[1], COPD represents a substantial public health problem. Acute exacerbations of COPD (AECOPD) are important drivers of mortality[2, 3] and reduced quality of life[4] in COPD patients and as the second most common reason for emergency hospital admission[5], they are also of great public health importance. Several studies[6–8] of AECOPD have been conducted in UK electronic healthcare records (EHR) which are becoming an increasingly important resource for evidence from real life research.

Data from primary care are used by organisations such as Public Health England (PHE) to compare data on AECOPD incidence and management across localities and by clinical commissioning groups to inform delivery of care and design of services. In addition, the recording of AECOPDs is important for clinicians as GPs need an easy and reliable way of accessing information on the timing and severity of previous AECOPD to tailor management programmes for their patients.

The investigation of AECOPD using EHR has so far been limited by the use of non-validated strategies to identify AECOPD events based on clinical experience. Previous studies used different combinations of drug therapy (for example, oral steroids and/or antibiotics)[7] and/or medical diagnosis codes. However, the validity of these approaches is not clear. Antibiotics may not be given if AECOPD are thought to be viral and, therefore, use of prescription of antibiotics alone may lead to misclassification of other diseases for AECOPD, particularly as up to 50% of AECOPD are known to be associated with a virus[9]. In addition, these prescriptions may be rescue packs intended for future use and may not represent individual acute events.

This study aimed to investigate a comprehensive set of pre-specified algorithms for the identification of AECOPD within UK primary care electronic healthcare records.

## Methods

### Data source

We used the Clinical Practice Research Datalink (CPRD), a large electronic database of UK general practice data that has been widely used for research. The Clinical Research Practice Datalink (CPRD)[10] is a large electronic database of primary care medical records. CPRD contains anonymised records for over 13 million patients, of which 4.4 million are currently registered with a practice that is contributing data to the CPRD, representing about 7% of the UK population. Data held include information on consultations, diagnoses, tests, referrals to secondary care and prescriptions from primary care as well as some lifestyle data. Around 60% of the patients included in the CPRD have been linked to hospital episode statistics data (HES).

### Codelist and algorithm development

Codelists (Read codes and product codes) were developed prior to the beginning of the study. Read codes are a hierarchical coding system of clinical terms used in the UK general practice which are entered into the GP software system and uploaded to the CPRD. Prescriptions for drugs are recorded in the CPRD as unique product codes. The codes used to construct AECOPD algorithms are available in the supplementary appendix (S2 File).

Strategies to ascertain AECOPD, translated into coding algorithms, were developed prior to the beginning of the study. These were based on both previous definitions that have been used in published papers, as well as definitions deemed to show high face validity. Face validity was determined after discussion between respiratory, primary care physicians with experience of UK primary care, and epidemiologists with experience in the design and analysis of studies using large UK primary care EHR databases. We used the August 2013 CPRD build and Read code dictionary. The fifteen algorithms are described in Table 1.

**Table 1:** Description of the algorithms tested.

Algorithm	Notes
1. Oral corticosteroid (OCS) prescription	For 5–14 days
2. Antibiotic prescription	For 5–14 days
3. Oral corticosteroid and antibiotic prescription	For 5–14 days, both on the same day
4. Exacerbation Symptom definition	Codes suggesting increase in two or more of: breathlessness, cough, or sputum volume and/or purulence
5. Exacerbation Symptom definition and oral corticosteroid prescription	Symptom definition the same as 4. Medical codes must have been on the same day as prescription. Duration of prescription was not limited.
6. Exacerbation Symptom definition and antibiotic prescription	Symptom definition the same as 4. Medical codes must have been on the same day as prescription. Duration of prescription was not limited.
7. Exacerbation Symptom definition and oral corticosteroid & antibiotic prescription	Symptom definition the same as 4. Medical codes must have been on the same day as prescription. Duration of prescription was not limited.
8. Lower respiratory tract infection (LRTI) code	Specifically excluding codes for pneumonia
9. LRTI code and oral corticosteroid prescription	Medical codes must have been on the same day as prescription. Duration of prescription was not limited.
10. LRTI code and antibiotic prescription	Medical codes must have been on the same day as prescription. Duration of prescription was not limited.
11. LRTI code and oral corticosteroid & antibiotic prescription	Medical codes must have been on the same day as prescription. Duration of prescription was not limited.
12. AECOPD code	
13. AECOPD code and oral corticosteroid prescription	Medical codes must have been on the same day as prescription. Duration of prescription was not limited.
14. AECOPD code and antibiotic prescription	Medical codes must have been on the same day as prescription. Duration of prescription was not limited.
15. AECOPD code and oral corticosteroid & antibiotic prescription	Medical codes must have been on the same day as prescription. Duration of prescription was not limited.

As prescription of rescue packs and acute codes used at annual reviews may be identified by our algorithms, we developed further codelists to identify consultations during which rescue packs were prescribed or annual reviews occurred.

### Study population

COPD patients were identified in the CPRD using a previously validated strategy[11]. For this analysis, we specifically defined COPD patients as having a record for a specific COPD Read code, history of current or past smoking, at least two prescriptions for COPD medicines (one within 4 weeks of the initial COPD Read code) and of age over 35 years at the time of the initial COPD Read code. Inclusion was further restricted to those patients whose GP practice last collection date was four months or less from the end of the study (August 2013) and were alive and registered at the GP practice at the time of the last CPRD data collection.

Patients were followed up from January 2004, date of COPD diagnosis or date of registration with an eligible practice, whichever was later and were followed up until August 2013, date of death, last collection date, or date of transfer out of an eligible GP practice, whichever was earlier. The fifteen pre-specified AECOPD algorithms were used to ascertain any potential AECOPD event which occurred during this time period.

For the validation purposes, potential AECOPD events identified via algorithms were further selected using stratified random sampling. This procedure was designed such that it would 1) select events randomly within algorithms, 2) maximise the amount of information available per questionnaire, and 3) select potential events from rarer algorithms preferentially over events from algorithms which had potential events which were more common. Briefly, 1600 patients were selected such that each algorithm was represented by potential AECOPD events in at least 100 patients. Up to 10 potential AECOPD events (up to 5 from a single algorithm) were then randomly selected from each patient’s individual pool of AECOPD events. This procedure ensured that several dates could be enquired about for each patient; that none of the definitions had no, or very few, potential AECOPD events in the final sample; and that the number of dates enquired about for each patient was not so high as to make response by the GP unlikely.

### Questionnaires

We sent a short questionnaire to GPs asking them to confirm whether their patients had AECOPD on the dates identified. GPs were allowed to respond with “Yes”, “No” or “Uncertain”. We also asked about any dates in the last 12 months on which the patient had an AECOPD, not already listed on the dates specified. Finally, we asked GPs to send copies of any relevant material, such as extracts from patient notes or hospital discharge letters. All material was anonymised by the CPRD before being returned to investigators. We sent two reminders to GP practices who did not initially respond.

### Outcome assessment

The reference standard for diagnosis of AECOPD was an independent review of all material from the GP (questionnaire and other relevant material) by two respiratory physicians. Each respiratory physician independently reviewed all available information before discussing disagreements. We calculated Cohen’s Kappa to assess inter-rater agreement. Information from CPRD on dates which the GP specified that their patient had an AECOPD, but which were not listed on the questionnaire, were also reviewed by a respiratory physician. These events were included in the analysis if they were judged to be an AECOPD. For potential AECOPD events which the GP responded with “uncertain”, we obtained and reviewed anonymised medical notes and information from the CPRD GP “free-text” field records corresponding to the appropriate date.

### Sample size

Assuming a conservative minimum of a 50% response rate and only 100 potential events identified per algorithm (50 AECOPD events per algorithm) in the final analytical sample, we calculated that the confidence intervals around example PPVs would be: 50% (95% CI, 35.5–64.5%); 70% (95% CI, 55.4–82.1%); 90% (95% CI, 78.2–96.7%).

### Analysis

The main outcome was positive predictive value (PPV). True positives were defined as events which were identified by the algorithm, sampled from the AECOPD pool and confirmed by the reference standard. False positives were defined as events which were identified by the algorithm, sampled from the AECOPD pool and not confirmed by the reference standard. PPV was calculated as: True positives / (True Positives + False Positives).

To estimate the sensitivity, we used a combination of algorithm and GP identified dates of AECOPD events in the last 12 months. True positives were defined as events (1) which were identified by the algorithm, sampled from the AECOPD pool and confirmed by the reference standard or (2) which were listed as additional events by the GP, which were also identified by algorithm but had not been sampled. False negatives were defined as events which were (1) listed by the GP as additional dates, but which were not identified by the algorithm or (2) as event dates which were identified and confirmed by the reference standard for other algorithm(s) only (i.e. confirmed events which were not in the AECOPD pool for that algorithm, whether sampled or not). For the analysis of sensitivity, events which occurred within two weeks of another event were considered part of the same episode. Sensitivity was calculated as: True Positives / (True Positives + False Negatives).

We used bootstrapping to obtain cluster-robust confidence intervals for PPV and sensitivity. We excluded events which were still “uncertain” after respiratory physician review. Events which occurred on the same day as annual reviews or rescue pack prescriptions were not included in the main analysis.

We repeated the analysis of PPV and sensitivity restricted to those patients for whom GPs sent additional information (patient notes and discharge summaries). In this group of patients, respiratory physicians who were assessing questionnaires would have been able to see information from several sources in order to reach a decision on whether they thought the patient had an AECOPD on the dates in question. We also repeated the analysis of PPV stratified by characteristics identified from the CPRD: age group, sex, smoking status, GOLD 2006 grade of airflow limitation[12], Medical Research Council (MRC) dyspnoea score[13], socioeconomic status[14], WHO Body Mass Index (BMI) category, previous record of asthma diagnosis, previous records of gastro-oesophageal reflux disease (GORD) diagnosis, and previous record of diagnosis for cardiovascular disease (either of myocardial infarction, angina or heart failure).

Finally, we assessed the PPV and sensitivity for several combinations of algorithms to identify AECOPD. Our strategy was to achieve an adequate sensitivity while maintaining a high PPV. Initially we combined algorithms which had the highest PPV (those with PPV>80%). We then added algorithms which had PPV>75% in order to improve sensitivity. We also calculated PPV and sensitivity using all of the algorithms.

### Ethics

Ethical approval was obtained from the London School of Hygiene and Tropical Medicine (LSHTM) Observational Research Ethics Committee (approval number 6481) and the Clinical Practice Research Datalink (CPRD) Independent Scientific Advisory Committee (ISAC) (approval number 13_116). Patient records and questionnaires were de-identified and anonymised by CPRD staff before being sent to the investigators.

## Results

### Patient characteristics

**Fig 1:**
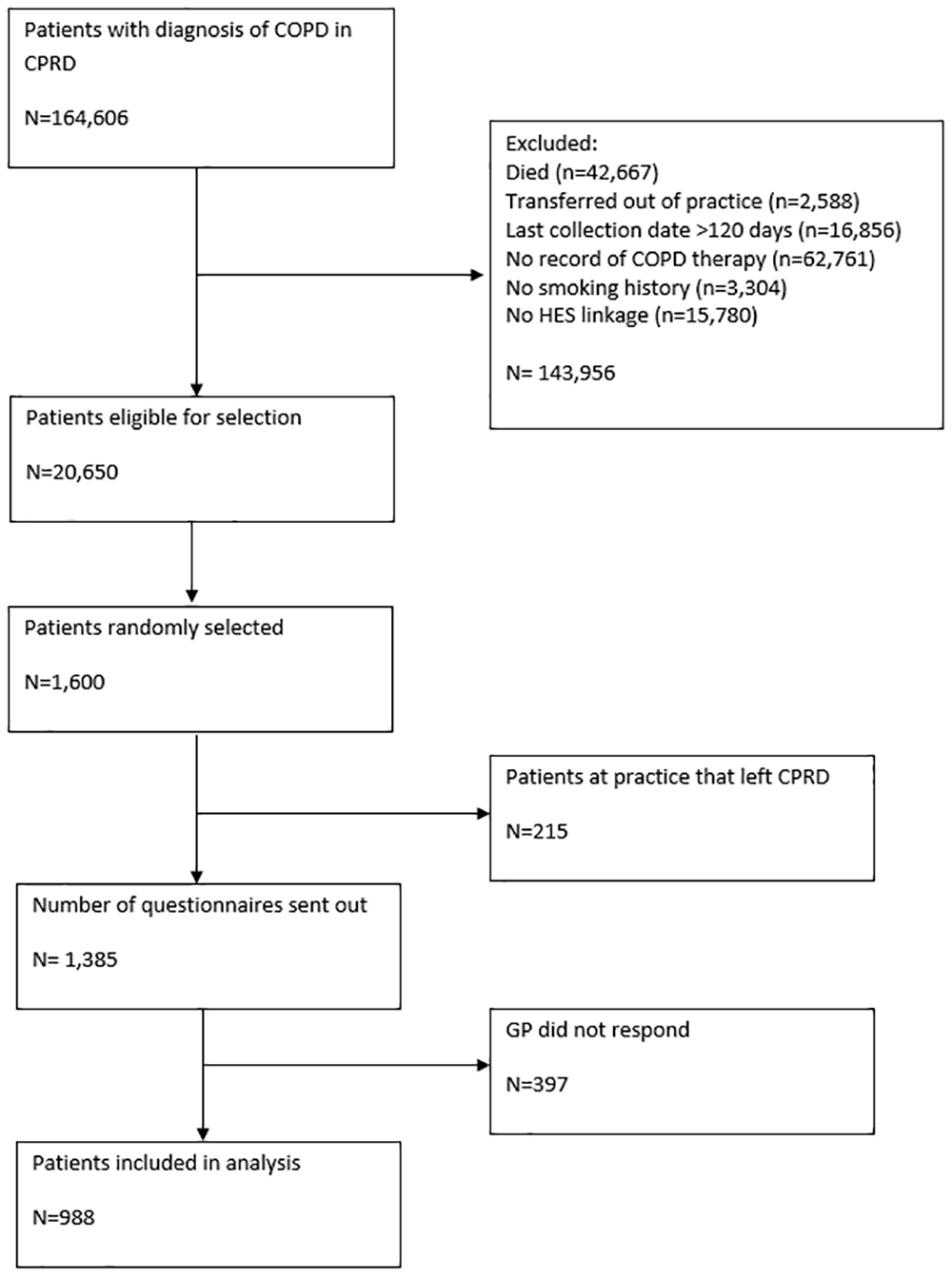
Patient flow through the study.

We selected 1600 patients for the study, of whom 215 had GP practices which had left the CPRD and were therefore excluded from the sampling frame (Fig 1). Our final study consisted of questionnaires related to the remaining 1385 patients. Of these 988 (71%) were returned by their GPs, representing 8258 potential AECOPD events. Characteristics of patients included in the study are detailed in Table 2. Mean age in our final sample of COPD patients was 62.4 years (SD, 10.6), 49% were male, 38% had severe or very severe airflow limitation (GOLD 2006 grades 3 or 4), 53% reported moderate/severe dyspnoea (MRC score of 3 or more), and 55% were current smokers. Restricting the sample to those dates which did not occur on annual review dates or dates of rescue pack prescriptions reduced the sample to 7136 events in 955 patients. Characteristics of patients whose GPs responded to the questionnaire were similar to those who did not, with the exception of socioeconomic status (Table A in S1 File). Patients whose GPs did not respond were on average more deprived than those whose GP responded. Details of the event flow through the study stratified by algorithm are presented in Table 3.

**Table 2:** Characteristics of the 988 patients included in the analysis.

Characteristic	n	% (N = 988)
**Age group**		
≤55	212	21.5
55 to 64	359	36.3
65 to 74	301	30.5
≥ 75	116	11.7
**Sex**		
Male	481	48.7
Female	507	51.3
**MRC breathlessness scale (N = 950)**		
≥3	449	47.3
< 3	501	52.7
**BMI**		
< 19	39	4.0
19–25	353	35.7
≥25	596	60.3
**Record of cardiovascular disease**		
No	731	74.0
Yes	257	26.0
**Record of asthma**		
No	482	48.8
Yes	506	51.2
**Record of GORD**		
No	729	73.8
Yes	259	26.2
**GOLD 2006 grade (N = 592)**		
1	76	12.8
2	285	48.1
3	185	31.3
4	46	7.8
**Smoking status**		
Ex-smoker	447	45.2
Current smoker	541	54.8
**Index of multiple deprivation quintile (N = 985)**		
1 (least deprived)	152	15.4
2	213	21.6
3	188	19.1
4	216	21.9
5 (most deprived)	216	21.9

**Table 3:** Flow of events through the study.

Algorithm	N events identified in the CPRD	N events sampled	N events from returned questionnaires	N events adjudicated for uncertain response	N events uncertain after respiratory physician review (% of those returned questionnaires)
**All**	261981	11697	8253	914	227 (2.8)
**1.OCS prescription for 5–14 days**	33898	1956	1285	120	32 (2.5)
**2.Antibiotic prescription for 5–14 days**	225761	9622	6283	809	208 (3.3)
**3.OCS and antibiotic prescription for 5–14 days**	22990	1374	919	72	22 (2.4)
**4. Symptom definition**	1745	462	341	11	2 (0.6)
**5. Symptom definition and OCS prescription**	553	232	156	6	1 (0.6)
**6. Symptom definition and antibiotic prescription**	165	132	108	5	0 (0)
**7. Symptom definition and OCS & antibiotic prescription**	142	112	90	3	0 (0)
**8. LRTI code**	60099	2753	1809	214	36 (2.0)
**9. LRTI code and OCS prescription**	53460	2488	1617	200	34 (2.1)
**10. LRTI code and antibiotic prescription**	9354	600	411	25	2 (0.5)
**11. LRTI code and OCS & antibiotic prescription**	8770	569	388	25	2 (0.5)
**12. AECOPD code**	20905	1371	966	21	0 (0)
**13. AECOPD code and OCS prescription**	15020	992	698	14	0 (0)
**14. AECOPD code and antibiotic prescription**	8571	674	466	11	0 (0)
**15. AECOPD code and OCS & antibiotic prescription**	7440	601	418	10	0 (0)

### PPV and sensitivity

Inter-rater agreement in outcome assessment was high. The respiratory physicians reviewing the questionnaires agreed for 92.5% of the potential AECOPD dates before discussion, and this resulted in a Cohen’s Kappa of 0.844. All disagreements were resolved by discussion between the two respiratory physicians and none were referred to a third physician. The PPVs and sensitivity of each algorithm are presented in Table 4. The algorithms with the higher PPVs (>80%) were those that used (1) an LRTI code along with either prescription of an antibiotic or a steroid or antibiotic and a steroid, and (2) AECOPD code either with or without prescription of antibiotics; and (3) the symptom definition with either prescription of OCS or antibiotics. The LRTI code alone (79.6%, 76.9–82.3%) and prescription of both antibiotics and OCS for 5–14 days (79.3%, 75.8–82.9%) had slightly lower PPVs. The symptom definition alone, prescription for 5–14 days of antibiotics and prescription of 5–14 days of OCS had poorer PPVs (60–73%).

**Table 4:** PPV and sensitivity for the algorithms.

Algorithm	N events identified in the CPRD	N events confirmed by reference standard	PPV (95% CI)	N events identified in the CPRD in last year	N extra events identified by other algorithms or GPs in last year	Sensitivity (95% CI)
**1.OCS prescription**	1152	841	73.0 (69.5–76.5)	164	379	30.2(25.8–34.6)
**2.Antibiotic prescription**	5840	3559	60.9 (59.0–62.9)	386	157	71.1 (66.8–75.4)
**3.OCS and antibiotic prescription**	823	653	79.3 (75.8–82.9)	133	410	24.5 (20.4–28.6)
**4. Symptom definition**	142	92	64.8 (56.2–73.3)	14	529	2.6 (1.1–4.0)
**5. Symptom definition and OCS prescription**	88	79	89.8 (82.9–96.7)	12	531	2.2 (0.9–3.6)
**6. Symptom definition and antibiotic prescription**	57	53	93.0 (85.6–100.0)	10	533	1.8 (0.6–3.1)
**7. Symptom definition and OCS & antibiotic prescription**	48	47	97.9 (94.5–100.0)	9	534	1.7 (0.5–2.9)
**8. LRTI code**	1745	1389	79.6 (76.9–82.3)	125	418	23.0 (19.2–26.8)
**9. LRTI code and OCS prescription**	1558	1268	81.4 (78.7–84.1)	108	435	19.9 (16.3–23.5)
**10. LRTI code and antibiotic prescription**	393	347	88.3 (84.4–92.2)	65	478	12.0 (9.3–14.7)
**11. LRTI code and OCS & antibiotic prescription**	371	327	88.1 (84.1–92.1)	62	481	11.4 (8.8–14.0)
**12. AECOPD code**	885	850	96.0 (94.5–97.6)	136	407	25.1 (20.9–29.2)
**13. AECOPD code and OCS prescription**	638	618	96.9 (95.4–98.3)	99	444	18.2 (14.6–21.8)
**14. AECOPD code and antibiotic prescription**	423	408	96.5 (94.5–98.4)	95	448	17.5 (13.8–21.2)
**15. AECOPD code and OCS & antibiotic prescription**	377	365	96.8 (95.0–98.6)	87	456	16.0 (12.6–19.5)

Antibiotics = selected antibiotics with clinical application in management of AECOPD

OCS = oral corticosteroids specific to AECOPD management

Sensitivity was low (<30%) for all algorithms except for prescription of an antibiotics course for 5–14 days (71.1%, 66.8–75.4%). More restrictive definitions had poorer sensitivity than those without any restriction. Sensitivity was particularly low for all of the algorithms which used respiratory symptoms.

Restricting the analysis to those patients for whom GPs sent supporting information resulted in slight increases in PPV for some algorithms (Table 5). This restriction also reduced the sensitivity for the use of OCS for 5–14 days to 22.7% (95% CI, 16.1–29.2%) from 30.2% (95% CI, 25.8–34.6%); the use of antibiotics for 5–14 days to 63.4% (95% CI, 55.4–71.4%) from 71.1% (95% CI, 66.8–75.4%); and the use of both antibiotics and OCS for 5–14 days to 18.6% (95% CI, 12.4–24.7%) from 24.5% (95% CI, 20.4–28.6%).

**Table 5:** PPV and sensitivity of the algorithms to identify AECOPD including only patients for whom additional information was available from their GP questionnaire.

Algorithm (inclusive definitions)	N events identified in the CPRD	N events confirmed by reference standard	PPV (95% CI)	N events identified in the CPRD in last year	N extra events identified by GPs in last year	Sensitivity (95% CI)
**1.OCS prescription**	367	265	72.2 (66.5–77.9)	44	150	22.7 (16.1–29.2)
**2.Antibiotic prescription**	2245	1376	61.3 (58.3–64.3)	123	71	63.4 (55.4–71.4)
**3.OCS and antibiotic prescription**	251	200	79.7 (73.5–85.8)	36	158	18.6 (12.4–24.7)
**4. Symptoms definition**	83	53	63.9 (52.7–75.0)	4	190	2.1 (0.1–4.0)
**5. Symptoms definition and OCSPrescription**	50	47	94.0 (88.0–100.0)	4	190	2.1 (0.1–4.0)
**6. Symptoms definition and antibiotic prescription**	36	34	94.4 (86.8–100.0)	3	191	1.6 (0.1–3.2)
**7. Symptoms definition and OCS & antibiotic prescription**	31	31	100.0 (88.8–100.0)	3	191	1.6 (0.1–3.2)
**8. LRTI code**	693	574	82.8 (78.8–86.9)	48	146	24.7 (18.8–30.7)
**9. LRTI code and OCS prescription**	621	525	84.5 (80.6–88.5)	40	154	20.6 (15.2–26.0)
**10. LRTI code and antibiotic prescription**	142	132	93.0 (88.3–97.6)	24	170	12.4 (7.8–16.9)
**11. LRTI code and OCS & antibiotic prescription**	129	119	92.2 (87.1–97.4)	21	173	10.8 (6.7–15.0)
**12. AECOPD code**	350	344	98.3 (96.9–99.6)	52	142	26.8 (19.7–33.9)
**13. AECOPD code and OCS prescription**	236	234	99.2 (98.1–100.0)	36	158	18.6 (12.4–24.7)
**14. AECOPD code and antibiotic prescription**	155	152	98.1 (96.0–100.0)	33	161	17.0 (10.8–23.2)
**15. AECOPD code and OCS & antibiotic prescription**	140	138	98.6 (96.8–100.0)	30	164	15.5 (9.7–21.2)

Antibiotics = selected antibiotics with clinical application in management of AECOPD

OCS = oral corticosteroids specific to AECOPD management

The analysis of PPV and sensitivity analyses were repeated for all event date including these dates occurring on annual COPD review and those with prescription for suspected rescue packs of OCS (Table B in S1 File). The PPVs stratified by patient demographic and disease severity characteristics are presented in the supplementary material (Tables C-L in S1 File). Briefly, PPVs for the OCS course for 5–14 days appeared to differ by some of the characteristics. PPV for the OCS course for 5–14 days was higher for patients with no or mild dyspnoea, without CVD co-morbidity, and for women.

The PPV and sensitivity for the composite strategies are presented in Table 6. Combining algorithms with PPV > 80% (5, 6, 8 or 12) resulted in a PPV of 88.1% (95% CI, 85.3–90.8) and a sensitivity of 51.6 (95% CI, 44.1–59.0). Using algorithms with a PPV >75% (3, 5, 6, 8 or 12) resulted a in very high PPV of 85.5% (95%CI, 82.7–88.3%) with a sensitivity of 62.9% (95%CI, 55.4–70.4%). Use of all pre-defined algorithms to identify AECOPD reduced the PPV to 63.8% (95%CI, 61.0–66.6%), but achieved a sensitivity of 88.1% (95%CI, 82.9–93.4%).

**Table 6:** PPV and sensitivity of composite strategies to identify AECOPD including only patients for whom additional information was available from their GP questionnaire.

Strategy	PPV (95% CI)	Sensitivity (95% CI)
**Algorithms with PPV > 80%** Algorithms 5, 6, 8 or 12: Symptom definition with prescription of antibiotic or OCS; or LRTI; or AECOPD code	88.1 (85.3–90.8)	51.6 (44.1–59.0)
**Algorithms with PPV > 75%** Algorithms 3, 5, 6, 8 or 12: Prescription of antibiotics and OCS for 5–14 days; or Symptom definition with prescription of antibiotic or OCS; or LRTI code; or AECOPD code	85.5 (82.7–88.3)	62.9 (55.4–70.4)
**All algorithms**	63.8 (61.0–66.6)	88.1 (82.9–93.4)

## Discussion

This is the first study to describe the recording of AECOPD by general practitioners in UK EHRs. Although the definitions used in future studies may depend on the individual needs and potential objectives, particularly with respect to the need for maximising either PPV or sensitivity, our recommendation for identifying AECOPD events in EHR is to use a composite of several of the definitions with higher PPV. To maximise sensitivity over PPV for identifying AECOPD in UK EHR, investigators would need to use prescription of antibiotics, as the PPV was low for this algorithm, this strategy is likely to misclassify many other infections as AECOPD. One recommended approach would be to use the following strategy that resulted in PPV of 86% and sensitivity of 63%: a combination of: (1) a medical diagnosis of LRTI or AECOPD, or (2) a prescription of COPD-specific antibiotic combined with OCS for 5–14 days, or (3) a record of two or more respiratory symptoms of AECOPD along with a prescription of COPD-specific antibiotics and/or OCS on the same day. These combined strategies should be used only after removing any AECOPD events occurring on the same date as codes suggestive of a visit for annual COPD review or provision of rescue packs for COPD-specific antibiotics or OCS. We do not recommend using definitions based on respiratory symptoms without COPD-specific antibiotics or OCS, or COPD-specific antibiotics or OCS without medical diagnosis of LRTI, AECOPD or respiratory symptoms due to mediocre PPVs. This has important implications as previous studies of AECOPD outcomes have used prescription of either antibiotics and or oral steroids to define AECOPD, and our findings suggest that this strategy may lead to a high level of misclassification of AECOPD events. Compared to previous studies, which have attempted to identify AECOPD in EHRs, we used a very specific list of antibiotics and OCS pertaining to management of AECOPD.

Having a validated definition of a COPD outcome, representing a substantial source of burden to patients and health-care providers, such as AECOPD is important. It provides a robust method for deriving statistics on AECOPD which can inform health-care service planning and evaluation of programs over time. In addition, as well as being a resource for “real life” observational studies, electronic healthcare records have the potential to be used in pragmatic clinical trials. This requires standardised and accurate definitions of exacerbations, and our research provides that.

Our findings illustrate that there are multiple strategies adopted by health care workers when recording AECOPD events in the UK EHR. Only about one half of the AECOPD events were recorded using a medical diagnosis code either for LRTI, AECOPD or respiratory symptoms, whilst the remaining events were recorded only as prescriptions of COPD-specific antibiotics and/or OCS. Even using all pre-defined algorithms, about 12% of AECOPD events failed to be captured (false negatives). We explored medical codes at these dates and did not find any leads allowing derivation of further algorithms. The most frequent events recorded on the AECOPD dates not captured by any algorithm included: “reviewed patient”, “home visit” or single symptoms. This heterogeneity makes ascertainment of AECOPD events challenging. We recommend that AECOPD events are recorded consistently by care providers, preferably using medical diagnosis codes stating AECOPD, and that these codes are recorded only at the time of acute events and not to record a historical number of prior episodes. This should be achieved through better education of prescribers, but also by improving health-care information systems to enable health care workers an easy and consistent way to record severity of AECOPD into EHRs, including patient reported AECOPD as milder events and retrieving hospital discharges for AECOPD. Moreover, AECOPD events which are treated by community COPD teams should be reported to GPs via linked health-care information systems to provide an integrated record of critical events. Ideally, GPs should be able to access AECOPD history of their patients with a “one-click” menu given its prognostic value, allowing for individually targeted treatment strategies for COPD patients at high risk of future events. One of the strengths of this study is the robust reference standard used to identify episodes of AECOPD though respiratory physicians independent adjudication of supplementary information from GPs as well as the anonymized “free-text” notes section from the CPRD.

Although we obtained information on AECOPD from GPs, there were still limitations to the available data. To maximize the rigor of the study, we used respiratory physician review of all available information as the reference standard, and we have presented a sensitivity analysis of only those events for which additional information was available. Although we had a reasonable response rate, GPs whose patients were more deprived were less likely to respond to our questionnaire and the extent to which the coding practices differ in association with patient deprivation level could not be determined. In addition, because we needed patients to be alive at the time of the study, our results may not be generalisable to those with the most severe COPD. Another limitation is that by using EHR to identify AECOPD, we will miss events which are self-managed by COPD patients, and therefore this study does not capture the full range of severity. Our results should therefore be interpreted as the accuracy of AECOPD events recorded by primary care clinicians. Our stratified analysis of PPV presented in the supplementary material showed that the algorithms based on symptom definitions and prescription of OCS alone for 5–14 days had different PPV depending on patient characteristics. These differences could potentially cause bias, however we do not recommend that prescription of OCS for 5–14 days alone is used to identify AECOPD, and the symptom-based definitions only contribute to a small number of the AECOPD events. In addition, the PPVs for definitions included in our recommended strategy (based on LRTI codes, AECOPD codes and prescription of both antibiotics and OCS) did not vary significantly depending on patient characteristics. Our recommended strategy for identifying AECOPD achieved a high PPV, however the sensitivity was lower, suggesting that although this strategy is valid it will tend to underestimate the number of events. One option for investigators wishing to assess the burden of AECOPD is to conduct an analysis using both a strategy with high PPV and one with high sensitivity in order to estimate a minimum and maximum number of events per patient. Our study was conducted in the UK, and this may limit generalisability of the results to EHR databases which collect data from other countries. Although we used definitions of AECOPD used in previous studies to develop our algorithms, it may be difficult to relate our findings to the validity of some previously used definitions. This is for two reasons, firstly, in order to achieve high validity, we used a narrow list of antibiotics in our algorithms. This is likely to have increased the PPV of our algorithms, and studies which used a broader list of antibiotics may have lower PPV for AECOPD identification. Secondly, poor reporting of previously used definitions of AECOPD mean that it is difficult to relate these to our current findings. One further limitation of the analysis presented here is that these results do not include hospital events, however this is the focus of a current study. This limitation should not affect the PPV, however this does mean that our estimates of sensitivity relate to events which are treated/recorded in primary care only and not the total number of AECOPD events.

We have validated strategies to identify AECOPD within electronic healthcare records, however our strategies may underestimate the total number of true AECOPD events. Our results should be used for future research studies and by public health bodies when identifying AECOPD in the UK. We found that some previously used definitions have low PPV. Our results also highlight the lack of standardisation of the recording of AECOPD in EHRs, and efforts should be made to standardise the recording of AECOPD within EHRs.

## Supporting Information

S1 File Tables A-L: Comparison of responders and non-responders and PPV for various algorithms stratified by patient characteristics. (DOCX)

S2 File Codes to construct AECOPD algorithms. (DOCX)
