# Supplementary material for: Validation of the Recording of Acute Exacerbations of COPD in UK Primary Care Electronic Healthcare Records
Source: PLoS One. 2016 Mar 9;11(3):e0151357. doi: 10.1371/journal.pone.0151357 (PMC4784784; doi:10.1371/journal.pone.0151357)
Supplement: S1 File — (DOCX) [file pone.0151357.s001.docx]

**Table A. Comparison of responders and non-responders**

| **Characteristic** | **Responder N** | **Responder %** | **non responder N** | **Non responder %** | **Chi^2^ p-value** |
| --- | --- | --- | --- | --- | --- |
| **Age group** |  |  |  |  | 0.073 |
| ≤55 | 212 | 21.5 | 111 | 28.0 |  |
| 55 to 64 | 359 | 36.3 | 133 | 33.5 |  |
| 65 to 74 | 301 | 30.5 | 107 | 27.0 |  |
| ≥ 75 | 116 | 11.7 | 46 | 11.6 |  |
| **Sex** |  |  |  |  | 0.850 |
| Male | 481 | 48.7 | 191 | 48.1 |  |
| Female | 507 | 51.3 | 206 | 51.9 |  |
| **MRC breathlessness scale** |  |  |  |  | 0.170 |
| ≥3 | 449 | 47.3 | 195 | 51.5 |  |
| < 3 | 501 | 52.7 | 184 | 48.6 |  |
| **BMI** |  |  |  |  | 0.520 |
| < 19 | 39 | 4.0 | 17 | 4.3 |  |
| 19 - 25 | 353 | 35.7 | 129 | 32.5 |  |
| ≥25 | 596 | 60.3 | 251 | 63.2 |  |
| **Record of cardiovascular disease** |  |  |  |  | 0.090 |
| No | 731 | 74.0 | 311 | 78.3 |  |
| Yes | 257 | 26.0 | 86 | 21.7 |  |
| **Record of asthma** |  |  |  |  | 0.380 |
| No | 482 | 48.8 | 204 | 51.4 |  |
| Yes | 506 | 51.2 | 193 | 48.6 |  |
| **Record of GORD** |  |  |  |  | 0.260 |
| No | 729 | 73.8 | 281 | 70.8 |  |
| Yes | 259 | 26.2 | 116 | 29.2 |  |
| **GOLD stage** |  |  |  |  | 0.330 |
| 1 | 76 | 12.8 | 39 | 16.7 |  |
| 2 | 285 | 48.1 | 113 | 48.5 |  |
| 3 | 185 | 31.3 | 61 | 26.2 |  |
| 4 | 46 | 7.8 | 20 | 8.6 |  |
| **Smoking status** |  |  |  |  | 0.220 |
| Ex-smoker | 447 | 45.2 | 194 | 48.9 |  |
| Current smoker | 541 | 54.8 | 203 | 51.1 |  |
| **Index of multiple deprivation quintile** |  |  |  |  | <0.001 |
| 1 (least deprived) | 152 | 15.4 | 32 | 8.1 |  |
| 2 | 213 | 21.6 | 55 | 14.0 |  |
| 3 | 188 | 19.1 | 64 | 16.2 |  |
| 4 | 216 | 21.9 | 100 | 25.4 |  |
| 5 (most deprived) | 216 | 21.9 | 143 | 36.3 |  |

**Table B. PPV and sensitivity for algorithms not excluding annual review dates and dates on which rescue packs were prescribed**

| **Algorithm** | **N events identified** | **N events confirmed by reference standard** | **PPV (95% CI)** | **N events identified in last year** | **N extra events identified by GPs in last year** | **Sensitivity (95% CI)** |
| --- | --- | --- | --- | --- | --- | --- |
| **1.OCS prescription for 5-14 days** | 1285 | 910 | 70.8 (68.3 - 73.3) | 180 | 465 | 27.9 (24.5 - 31.5) |
| **2.Antibiotic prescription for 5-14 days** | 6283 | 3796 | 60.4 (59.2 - 61.6) | 426 | 219 | 66.1 (62.3 - 69.7) |
| **3.OCS and antibiotic prescription for 5-14 days** | 919 | 705 | 76.7 (73.8 - 79.4) | 142 | 503 | 22.0 (18.9 - 25.4) |
| **4. Symptom definition** | 341 | 137 | 40.2 (34.9 - 45.6) | 16 | 629 | 2.5 (1.4 - 4.0) |
| **5. Symptom definition and OCS prescription** | 156 | 106 | 68.0 (60.0 - 75.2) | 14 | 631 | 2.2 (1.2 - 3.6) |
| **6. Symptom definition and antibiotic prescription** | 108 | 74 | 68.5 (58.9 - 77.1) | 11 | 634 | 1.7 (0.9 - 3.0) |
| **7. Symptom definition and OCS & antibiotic prescription** | 90 | 64 | 71.1 (60.6 - 80.2) | 10 | 635 | 1.6 (0.8 - 2.8) |
| **8. LRTI code** | 1809 | 1435 | 79.3 (77.4 - 81.2) | 132 | 513 | 20.5 (17.4 - 23.8) |
| **9. LRTI code and OCS prescription** | 1617 | 1311 | 81.1 (79.1 – 83.0) | 116 | 529 | 18.0 (15.1 - 21.2) |
| **10. LRTI code and antibiotic prescription** | 411 | 362 | 88.1 (84.6 - 91.1) | 73 | 572 | 11.3 (9.0 - 14.0) |
| **11. LRTI code and OCS & antibiotic prescription** | 388 | 342 | 88.1 (84.5 - 91.2) | 70 | 575 | 10.9 (8.6 - 13.5) |
| **12. AECOPD code** | 966 | 905 | 93.7 (92.0 - 95.1) | 147 | 498 | 22.8 (19.6 - 26.2) |
| **13. AECOPD code and OCS prescription** | 698 | 667 | 95.6 (93.8 – 97.0) | 105 | 540 | 16.3 (13.5 - 19.4) |
| **14. AECOPD code and antibiotic prescription** | 466 | 443 | 95.1 (92.7 - 96.9) | 98 | 547 | 15.2 (12.5 - 18.2) |
| **15. AECOPD code and OCS & antibiotic prescription** | 418 | 398 | 95.2 (92.7 - 97.1) | 90 | 555 | 14.0 (11.4 - 16.9) |

**Table C. PPVs for algorithms stratified by MRC breathlessness score**

| **Algorithm** | **N events identified** | **N events confirmed by reference standard** | **PPV (95% CI)** | **N events identified** | **N events confirmed by reference standard** | **PPV (95% CI)** |
| --- | --- | --- | --- | --- | --- | --- |
|  | **MRC ≥3** |  |  | **MRC < 3** |  |  |
| **1.OCS prescription** | 535 | 364 | 68.0 (63.9 - 72.0) | 569 | 438 | 77.0 (73.3 - 80.4) |
| **2.Antibiotic prescription** | 2626 | 1589 | 60.5 (58.6 - 62.4) | 2994 | 1836 | 61.3 (59.6 - 63.1) |
| **3.OCS and antibiotic prescription** | 371 | 285 | 76.8 (72.2 - 81.0) | 422 | 340 | 80.6 (76.5 - 84.2) |
| **4. Symptom definition** | 73 | 49 | 67.1 (55.1 - 77.7) | 61 | 36 | 59.0 (45.7 - 71.4) |
| **5. Symptom definition and OCS prescription** | 43 | 39 | 90.7 (77.9 - 97.4) | 39 | 34 | 87.2 (72.6 - 95.7) |
| **6. Symptom definition and antibiotic prescription** | 30 | 28 | 93.3 (77.9 - 99.2) | 22 | 20 | 90.9 (70.8 - 98.9) |
| **7. Symptom definition and OCS & antibiotic prescription** | 25 | 25 | 100.0 (86.3 - 100.0) | 19 | 18 | 94.7 (74.0 - 99.9) |
| **8. LRTI code** | 779 | 608 | 78.0 (75.0 - 80.9) | 892 | 722 | 80.9 (78.2 - 83.5) |
| **9. LRTI code and OCS prescription** | 687 | 541 | 78.7 (75.5 - 81.8) | 803 | 671 | 83.6 (80.8 - 86.1) |
| **10. LRTI code and antibiotic prescription** | 175 | 151 | 86.3 (80.3 - 91.0) | 191 | 171 | 89.5 (84.3 - 93.5) |
| **11. LRTI code and OCS & antibiotic prescription** | 160 | 137 | 85.6 (79.2 - 90.7) | 186 | 166 | 89.2 (83.9 - 93.3) |
| **12. AECOPD code** | 459 | 442 | 96.3 (94.1 - 97.8) | 411 | 393 | 95.6 (93.2 - 97.4) |
| **13. AECOPD code and OCS prescription** | 304 | 294 | 96.7 (94.0 - 98.4) | 325 | 315 | 96.9 (94.4 - 98.5) |
| **14. AECOPD code and antibiotic prescription** | 183 | 176 | 96.2 (92.3 - 98.4) | 232 | 224 | 96.6 (93.3 - 98.5) |
| **15. AECOPD code and OCS & antibiotic prescription** | 164 | 157 | 95.7 (91.4 - 98.3) | 206 | 201 | 97.6 (94.4 - 99.2) |

**Table D. PPVs for algorithms stratified by deprivation**

| **Algorithm** | **N events identified** | **N events confirmed by reference standard** | **PPV (95% CI)** | **N events identified** | **N events confirmed by reference standard** | **PPV (95% CI)** |
| --- | --- | --- | --- | --- | --- | --- |
|  | **Less deprived** | | | **More deprived** | | |
| **1.OCS prescription** | 612 | 453 | 74.0 (70.4 - 77.5) | 540 | 388 | 71.9 (67.9 - 75.6) |
| **2.Antibiotic prescription** | 3160 | 1955 | 61.9 (60.1 - 63.6) | 2680 | 1604 | 59.9 (58.0 - 61.7) |
| **3.OCS and antibiotic prescription** | 441 | 359 | 81.4 (77.5 - 84.9) | 382 | 294 | 77.0 (72.4 - 81.1) |
| **4. Symptom definition** | 60 | 32 | 53.3 (40.0 - 66.3) | 82 | 60 | 73.2 (62.2 - 82.4) |
| **5. Symptom definition and OCS prescription** | 30 | 24 | 80.0 (61.4 - 92.3) | 58 | 55 | 94.8 (85.6 - 98.9) |
| **6. Symptom definition and antibiotic prescription** | 12 | 10 | 83.3 (51.6 - 97.9) | 45 | 43 | 95.6 (84.9 - 99.5) |
| **7. Symptom definition and OCS & antibiotic prescription** | 9 | 8 | 88.9 (51.8 - 99.7) | 39 | 39 | 100.0 (91.0 - 100.0) |
| **8. LRTI code** | 909 | 714 | 78.5 (75.7 - 81.2) | 836 | 675 | 80.7 (77.9 - 83.4) |
| **9. LRTI code and OCS prescription** | 804 | 649 | 80.7 (77.8 - 83.4) | 754 | 619 | 82.1 (79.2 - 84.8) |
| **10. LRTI code and antibiotic prescription** | 191 | 168 | 88.0 (82.5 - 92.2) | 202 | 179 | 88.6 (83.4 - 92.6) |
| **11. LRTI code and OCS & antibiotic prescription** | 177 | 155 | 87.6 (81.8 - 92.0) | 194 | 172 | 88.7 (83.3 - 92.8) |
| **12. AECOPD code** | 502 | 476 | 94.8 (92.5 - 96.6) | 383 | 374 | 97.7 (95.6 - 98.9) |
| **13. AECOPD code and OCS prescription** | 365 | 350 | 95.9 (93.3 - 97.7) | 273 | 268 | 98.2 (95.8 - 99.4) |
| **14. AECOPD code and antibiotic prescription** | 243 | 231 | 95.1 (91.5 - 97.4) | 180 | 177 | 98.3 (95.2 - 99.7) |
| **15. AECOPD code and OCS & antibiotic prescription** | 214 | 205 | 95.8 (92.2 - 98.1) | 163 | 160 | 98.2 (94.7 - 99.6) |

**Table E. PPVs for algorithms stratified by GOLD stage**

| **Algorithm** | **N events identified** | **N events confirmed by reference standard** | **PPV (95% CI)** | **N events identified** | **N events confirmed by reference standard** | **PPV (95% CI)** |
| --- | --- | --- | --- | --- | --- | --- |
|  | **GOLD 1-2** | | | **GOLD 3-4** | | |
| **1.OCS prescription** | 839 | 621 | 74.0 (70.9 - 77.0) | 313 | 220 | 70.3 (64.9 - 75.3) |
| **2.Antibiotic prescription** | 4484 | 2672 | 59.6 (58.1 - 61.0) | 1356 | 887 | 65.4 (62.8 - 67.9) |
| **3.OCS and antibiotic prescription** | 608 | 487 | 80.1 (76.7 - 83.2) | 215 | 166 | 77.2 (71.0 - 82.6) |
| **4. Symptom definition** | 103 | 62 | 60.2 (50.1 - 69.7) | 39 | 30 | 76.9 (60.7 - 88.9) |
| **5. Symptom definition and OCS prescription** | 62 | 55 | 88.7 (78.1 - 95.3) | 26 | 24 | 92.3 (74.9 - 99.1) |
| **6. Symptom definition and antibiotic prescription** | 41 | 40 | 97.6 (87.1 - 99.9) | 16 | 13 | 81.3 (54.4 - 96.0) |
| **7. Symptom definition and OCS & antibiotic prescription** | 35 | 35 | 100.0 (90.0 - 100.0) | 13 | 12 | 92.3 (64.0 - 99.8) |
| **8. LRTI code** | 1372 | 1075 | 78.4 (76.1 - 80.5) | 373 | 314 | 84.2 (80.1 - 87.7) |
| **9. LRTI code and OCS prescription** | 1229 | 986 | 80.2 (77.9 - 82.4) | 329 | 282 | 85.7 (81.5 - 89.3) |
| **10. LRTI code and antibiotic prescription** | 298 | 263 | 88.3 (84.0 - 91.7) | 95 | 84 | 88.4 (80.2 - 94.1) |
| **11. LRTI code and OCS & antibiotic prescription** | 285 | 251 | 88.1 (83.7 - 91.6) | 86 | 76 | 88.4 (79.7 - 94.3) |
| **12. AECOPD code** | 617 | 594 | 96.3 (94.5 - 97.6) | 268 | 256 | 95.5 (92.3 - 97.7) |
| **13. AECOPD code and OCS prescription** | 445 | 432 | 97.1 (95.1 - 98.4) | 193 | 186 | 96.4 (92.7 - 98.5) |
| **14. AECOPD code and antibiotic prescription** | 304 | 294 | 96.7 (94.0 - 98.4) | 119 | 114 | 95.8 (90.5 - 98.6) |
| **15. AECOPD code and OCS & antibiotic prescription** | 270 | 263 | 97.4 (94.7 - 99.0) | 107 | 102 | 95.3 (89.4 - 98.5) |

**Table F. PPVs for algorithms stratified by record for asthma**

| **Algorithm** | **N events identified** | **N events confirmed by reference standard** | **PPV (95% CI)** | **N events identified** | **N events confirmed by reference standard** | **PPV (95% CI)** |
| --- | --- | --- | --- | --- | --- | --- |
|  | **Asthma record** | | | **No asthma record** | | |
| **1.OCS prescription** | 639 | 468 | 73.2 (69.6 - 76.6) | 513 | 373 | 72.7 (68.6 - 76.5) |
| **2.Antibiotic prescription** | 3085 | 1897 | 61.5 (59.7 - 63.2) | 2755 | 1662 | 60.3 (58.5 - 62.2) |
| **3.OCS and antibiotic prescription** | 444 | 359 | 80.9 (76.9 - 84.4) | 379 | 294 | 77.6 (73.0 - 81.7) |
| **4. Symptom definition** | 80 | 56 | 70.0 (58.7 - 79.7) | 62 | 36 | 58.1 (44.8 - 70.5) |
| **5. Symptom definition and OCS prescription** | 51 | 47 | 92.2 (81.1 - 97.8) | 37 | 32 | 86.5 (71.2 - 95.5) |
| **6. Symptom definition and antibiotic prescription** | 36 | 34 | 94.4 (81.3 - 99.3) | 21 | 19 | 90.5 (69.6 - 98.8) |
| **7. Symptom definition and OCS & antibiotic prescription** | 30 | 29 | 96.7 (82.8 - 99.9) | 18 | 18 | 100.0 (81.5 - 100.0) |
| **8. LRTI code** | 925 | 751 | 81.2 (78.5 - 83.7) | 820 | 638 | 77.8 (74.8 - 80.6) |
| **9. LRTI code and OCS prescription** | 832 | 685 | 82.3 (79.6 - 84.9) | 726 | 583 | 80.3 (77.2 - 83.1) |
| **10. LRTI code and antibiotic prescription** | 233 | 208 | 89.3 (84.6 - 92.9) | 160 | 139 | 86.9 (80.6 - 91.7) |
| **11. LRTI code and OCS & antibiotic prescription** | 217 | 193 | 88.9 (84.0 - 92.8) | 154 | 134 | 87.0 (80.7 - 91.9) |
| **12. AECOPD code** | 481 | 454 | 94.4 (91.9 - 96.3) | 404 | 396 | 98.0 (96.1 - 99.1) |
| **13. AECOPD code and OCS prescription** | 339 | 324 | 95.6 (92.8 - 97.5) | 299 | 294 | 98.3 (96.1 - 99.5) |
| **14. AECOPD code and antibiotic prescription** | 224 | 211 | 94.2 (90.3 - 96.9) | 199 | 197 | 99.0 (96.4 - 99.9) |
| **15. AECOPD code and OCS & antibiotic prescription** | 192 | 182 | 94.8 (90.6 - 97.5) | 185 | 183 | 98.9 (96.1 - 99.9) |

**Table G. PPVs for algorithms stratified by record for GORD**

| **Algorithm** | **N events identified** | **N events confirmed by reference standard** | **PPV (95% CI)** | **N events identified** | **N events confirmed by reference standard** | **PPV (95% CI)** |
| --- | --- | --- | --- | --- | --- | --- |
|  | **GORD record** | | | **No GORD record** | | |
| **1.OCS prescription** | 272 | 199 | 73.2 (67.5 - 78.3) | 880 | 642 | 73.0 (69.9 - 75.9) |
| **2.Antibiotic prescription** | 1623 | 941 | 58.0 (55.5 - 60.4) | 4217 | 2618 | 62.1 (60.6 - 63.5) |
| **3.OCS and antibiotic prescription** | 197 | 155 | 78.7 (72.3 - 84.2) | 626 | 498 | 79.6 (76.2 - 82.6) |
| **4. Symptom definition** | 37 | 22 | 59.5 (42.1 - 75.2) | 105 | 70 | 66.7 (56.8 - 75.6) |
| **5. Symptom definition and OCS prescription** | 22 | 19 | 86.4 (65.1 - 97.1) | 66 | 60 | 90.9 (81.3 - 96.6) |
| **6. Symptom definition and antibiotic prescription** | 13 | 11 | 84.6 (54.6 - 98.1) | 44 | 42 | 95.5 (84.5 - 99.4) |
| **7. Symptom definition and OCS & antibiotic prescription** | 12 | 11 | 91.7 (61.5 - 99.8) | 36 | 36 | 100.0 (90.3 - 100.0) |
| **8. LRTI code** | 484 | 369 | 76.2 (72.2 - 80.0) | 1261 | 1020 | 80.9 (78.6 - 83.0) |
| **9. LRTI code and OCS prescription** | 424 | 337 | 79.5 (75.3 - 83.2) | 1134 | 931 | 82.1 (79.7 - 84.3) |
| **10. LRTI code and antibiotic prescription** | 90 | 78 | 86.7 (77.9 - 92.9) | 303 | 269 | 88.8 (84.7 - 92.1) |
| **11. LRTI code and OCS & antibiotic prescription** | 82 | 71 | 86.6 (77.3 - 93.1) | 289 | 256 | 88.6 (84.3 - 92.0) |
| **12. AECOPD code** | 235 | 223 | 94.9 (91.3 - 97.3) | 650 | 627 | 96.5 (94.7 - 97.7) |
| **13. AECOPD code and OCS prescription** | 161 | 155 | 96.3 (92.1 - 98.6) | 477 | 463 | 97.1 (95.1 - 98.4) |
| **14. AECOPD code and antibiotic prescription** | 103 | 96 | 93.2 (86.5 - 97.2) | 320 | 312 | 97.5 (95.1 - 98.9) |
| **15. AECOPD code and OCS & antibiotic prescription** | 91 | 86 | 94.5 (87.6 - 98.2) | 286 | 279 | 97.6 (95.0 - 99.0) |

**Table H. PPVs for algorithms stratified by record for CVD**

| **Algorithm** | **N events identified** | **N events confirmed by reference standard** | **PPV (95% CI)** | **N events identified** | **N events confirmed by reference standard** | **PPV (95% CI)** |
| --- | --- | --- | --- | --- | --- | --- |
|  | **CVD record** | | | **No CVD record** | | |
| **1.OCS prescription** | 296 | 198 | 66.9 (61.2 - 72.2) | 856 | 643 | 75.1 (72.1 - 78.0) |
| **2.Antibiotic prescription** | 1536 | 924 | 60.2 (57.7 - 62.6) | 4304 | 2635 | 61.2 (59.7 - 62.7) |
| **3.OCS and antibiotic prescription** | 198 | 153 | 77.3 (70.8 - 82.9) | 625 | 500 | 80.0 (76.6 - 83.1) |
| **4. Symptom definition** | 44 | 28 | 63.6 (47.8 - 77.6) | 98 | 64 | 65.3 (55.0 - 74.6) |
| **5. Symptom definition and OCS prescription** | 27 | 25 | 92.6 (75.7 - 99.1) | 61 | 54 | 88.5 (77.8 - 95.3) |
| **6. Symptom definition and antibiotic prescription** | 15 | 15 | 100.0 (78.2 - 100.0) | 42 | 38 | 90.5 (77.4 - 97.3) |
| **7. Symptom definition and OCS & antibiotic prescription** | 15 | 15 | 100.0 (78.2 - 100.0) | 33 | 32 | 97.0 (84.2 - 99.9) |
| **8. LRTI code** | 478 | 365 | 76.4 (72.3 - 80.1) | 1267 | 1024 | 80.8 (78.5 - 83.0) |
| **9. LRTI code and OCS prescription** | 415 | 325 | 78.3 (74.0 - 82.2) | 1143 | 943 | 82.5 (80.2 - 84.7) |
| **10. LRTI code and antibiotic prescription** | 106 | 92 | 86.8 (78.8 - 92.6) | 287 | 255 | 88.9 (84.6 - 92.2) |
| **11. LRTI code and OCS & antibiotic prescription** | 100 | 86 | 86.0 (77.6 - 92.1) | 271 | 241 | 88.9 (84.6 - 92.4) |
| **12. AECOPD code** | 245 | 234 | 95.5 (92.1 - 97.7) | 640 | 616 | 96.3 (94.5 - 97.6) |
| **13. AECOPD code and OCS prescription** | 159 | 152 | 95.6 (91.1 - 98.2) | 479 | 466 | 97.3 (95.4 - 98.5) |
| **14. AECOPD code and antibiotic prescription** | 107 | 103 | 96.3 (90.7 - 99.0) | 316 | 305 | 96.5 (93.9 - 98.2) |
| **15. AECOPD code and OCS & antibiotic prescription** | 95 | 91 | 95.8 (89.6 - 98.8) | 282 | 274 | 97.2 (94.5 - 98.8) |

**Table I. PPVs for algorithms stratified by BMI**

| **Algorithm** | **N events identified** | **N events confirmed by reference standard** | **PPV (95% CI)** | **N events identified** | **N events confirmed by reference standard** | | **PPV (95% CI)** | **N events identified** | **N events confirmed by reference standard** | **PPV (95% CI)** |
| --- | --- | --- | --- | --- | --- | --- | --- | --- | --- | --- |
|  | **BMI <19** | | | **BMI 19-25** | | | | **BMI ≥25** | | |
| **1.OCS prescription** | 54 | 40 | 74.1 (60.3 - 85.0) | 445 | 344 | 77.3 (73.1 - 81.1) | | 653 | 457 | 70.0 (66.3 - 73.5) |
| **2.Antibiotic prescription** | 252 | 169 | 67.1 (60.9 - 72.8) | 2081 | 1330 | 63.9 (61.8 - 66.0) | | 3507 | 2060 | 58.7 (57.1 - 60.4) |
| **3.OCS and antibiotic prescription** | 41 | 34 | 82.9 (67.9 - 92.8) | 307 | 255 | 83.1 (78.4 - 87.1) | | 475 | 364 | 76.6 (72.6 - 80.4) |
| **4. Symptom definition** | 10 | 6 | 60.0 (26.2 - 87.8) | 34 | 23 | 67.6 (49.5 - 82.6) | | 98 | 63 | 64.3 (54.0 - 73.7) |
| **5. Symptom definition and OCS prescription** | 5 | 5 | 100.0 (47.8 - 100.0) | 21 | 18 | 85.7 (63.7 - 97.0) | | 62 | 56 | 90.3 (80.1 - 96.4) |
| **6. Symptom definition and antibiotic prescription** | 7 | 5 | 71.4 (29.0 - 96.3) | 10 | 9 | 90.0 (55.5 - 99.7) | | 40 | 39 | 97.5 (86.8 - 99.9) |
| **7. Symptom definition and OCS & antibiotic prescription** | 4 | 4 | 100.0 (39.8 - 100.0) | 9 | 8 | 88.9 (51.8 - 99.7) | | 35 | 35 | 100.0 (90.0 - 100.0) |
| **8. LRTI code** | 76 | 57 | 75.0 (63.7 - 84.2) | 541 | 454 | 83.9 (80.5 - 86.9) | | 1128 | 878 | 77.8 (75.3 - 80.2) |
| **9. LRTI code and OCS prescription** | 71 | 54 | 76.1 (64.5 - 85.4) | 481 | 413 | 85.9 (82.4 - 88.9) | | 1006 | 801 | 79.6 (77.0 - 82.1) |
| **10. LRTI code and antibiotic prescription** | 17 | 11 | 64.7 (38.3 - 85.8) | 134 | 128 | 95.5 (90.5 - 98.3) | | 242 | 208 | 86.0 (80.9 - 90.1) |
| **11. LRTI code and OCS & antibiotic prescription** | 16 | 10 | 62.5 (35.4 - 84.8) | 122 | 116 | 95.1 (89.6 - 98.2) | | 233 | 201 | 86.3 (81.2 - 90.4) |
| **12. AECOPD code** | 40 | 39 | 97.5 (86.8 - 99.9) | 376 | 360 | 95.7 (93.2 - 97.5) | | 469 | 451 | 96.2 (94.0 - 97.7) |
| **13. AECOPD code and OCS prescription** | 29 | 28 | 96.6 (82.2 - 99.9) | 283 | 275 | 97.2 (94.5 - 98.8) | | 326 | 315 | 96.6 (94.0 - 98.3) |
| **14. AECOPD code and antibiotic prescription** | 18 | 18 | 100.0 (81.5 - 100.0) | 183 | 180 | 98.4 (95.3 - 99.7) | | 222 | 210 | 94.6 (90.7 - 97.2) |
| **15. AECOPD code and OCS & antibiotic prescription** | 16 | 16 | 100.0 (79.4 - 100.0) | 167 | 164 | 98.2 (94.8 - 99.6) | | 194 | 185 | 95.4 (91.4 - 97.9) |

**Table J. PPVs for algorithms stratified by sex**

| **Algorithm** | **N events identified** | **N events confirmed by reference standard** | **PPV (95% CI)** | **N events identified** | **N events confirmed by reference standard** | **PPV (95% CI)** |
| --- | --- | --- | --- | --- | --- | --- |
|  | **Female** | | | **Male** | | |
| **1.OCS prescription** | 609 | 469 | 77.0 (73.5 - 80.3) | 536 | 367 | 68.5 (64.3 - 72.4) |
| **2.Antibiotic prescription** | 3015 | 1843 | 61.1 (59.4 - 62.9) | 2777 | 1687 | 60.7 (58.9 - 62.6) |
| **3.OCS and antibiotic prescription** | 433 | 352 | 81.3 (77.3 - 84.9) | 385 | 296 | 76.9 (72.3 - 81.0) |
| **4. Symptom definition** | 66 | 41 | 62.1 (49.3 - 73.8) | 75 | 51 | 68.0 (56.2 - 78.3) |
| **5. Symptom definition and OCS prescription** | 40 | 34 | 85.0 (70.2 - 94.3) | 48 | 45 | 93.8 (82.8 - 98.7) |
| **6. Symptom definition and antibiotic prescription** | 28 | 25 | 89.3 (71.8 - 97.7) | 29 | 28 | 96.6 (82.2 - 99.9) |
| **7. Symptom definition and OCS & antibiotic prescription** | 23 | 22 | 95.7 (78.1 - 99.9) | 25 | 25 | 100.0 (86.3 - 100.0) |
| **8. LRTI code** | 913 | 732 | 80.2 (77.4 - 82.7) | 818 | 646 | 79.0 (76.0 - 81.7) |
| **9. LRTI code and OCS prescription** | 811 | 662 | 81.6 (78.8 - 84.2) | 736 | 597 | 81.1 (78.1 - 83.9) |
| **10. LRTI code and antibiotic prescription** | 217 | 193 | 88.9 (84.0 - 92.8) | 175 | 153 | 87.4 (81.6 - 92.0) |
| **11. LRTI code and OCS & antibiotic prescription** | 203 | 181 | 89.2 (84.1 - 93.1) | 167 | 145 | 86.8 (80.7 - 91.6) |
| **12. AECOPD code** | 456 | 439 | 96.3 (94.1 - 97.8) | 420 | 402 | 95.7 (93.3 - 97.4) |
| **13. AECOPD code and OCS prescription** | 326 | 316 | 96.9 (94.4 - 98.5) | 307 | 297 | 96.7 (94.1 - 98.4) |
| **14. AECOPD code and antibiotic prescription** | 224 | 218 | 97.3 (94.3 - 99.0) | 196 | 187 | 95.4 (91.5 - 97.9) |
| **15. AECOPD code and OCS & antibiotic prescription** | 195 | 190 | 97.4 (94.1 - 99.2) | 179 | 172 | 96.1 (92.1 - 98.4) |

**Table K. PPVs for algorithms stratified by smoking status**

| **Algorithm** | **N events identified** | **N events confirmed by reference standard** | **PPV (95% CI)** | **N events identified** | **N events confirmed by reference standard** | **PPV (95% CI)** |
| --- | --- | --- | --- | --- | --- | --- |
|  | **Ex-smoker** | | | **Current smoker** | | |
| **1.OCS prescription** | 490 | 344 | 70.2 (65.9 - 74.2) | 662 | 497 | 75.1 (71.6 - 78.3) |
| **2.Antibiotic prescription** | 2621 | 1601 | 61.1 (59.2 - 63.0) | 3219 | 1958 | 60.8 (59.1 - 62.5) |
| **3.OCS and antibiotic prescription** | 345 | 271 | 78.6 (73.8 - 82.8) | 478 | 382 | 79.9 (76.0 - 83.4) |
| **4. Symptom definition** | 58 | 45 | 77.6 (64.7 - 87.5) | 84 | 47 | 56.0 (44.7 - 66.8) |
| **5. Symptom definition and OCS prescription** | 39 | 37 | 94.9 (82.7 - 99.4) | 49 | 42 | 85.7 (72.8 - 94.1) |
| **6. Symptom definition and antibiotic prescription** | 27 | 24 | 88.9 (70.8 - 97.6) | 30 | 29 | 96.7 (82.8 - 99.9) |
| **7. Symptom definition and OCS & antibiotic prescription** | 21 | 20 | 95.2 (76.2 - 99.9) | 27 | 27 | 100.0 (87.2 - 100.0) |
| **8. LRTI code** | 805 | 663 | 82.4 (79.5 - 84.9) | 940 | 726 | 77.2 (74.4 - 79.9) |
| **9. LRTI code and OCS prescription** | 722 | 607 | 84.1 (81.2 - 86.7) | 836 | 661 | 79.1 (76.1 - 81.8) |
| **10. LRTI code and antibiotic prescription** | 166 | 143 | 86.1 (79.9 - 91.0) | 227 | 204 | 89.9 (85.2 - 93.5) |
| **11. LRTI code and OCS & antibiotic prescription** | 161 | 139 | 86.3 (80.0 - 91.2) | 210 | 188 | 89.5 (84.6 - 93.3) |
| **12. AECOPD code** | 406 | 392 | 96.6 (94.3 - 98.1) | 479 | 458 | 95.6 (93.4 - 97.3) |
| **13. AECOPD code and OCS prescription** | 283 | 273 | 96.5 (93.6 - 98.3) | 355 | 345 | 97.2 (94.9 - 98.6) |
| **14. AECOPD code and antibiotic prescription** | 171 | 164 | 95.9 (91.7 - 98.3) | 252 | 244 | 96.8 (93.8 - 98.6) |
| **15. AECOPD code and OCS & antibiotic prescription** | 147 | 140 | 95.2 (90.4 - 98.1) | 230 | 225 | 97.8 (95.0 - 99.3) |

**Table L. PPVs for algorithms stratified by age group**

| **Algorithm** | **N events identified** | **N events confirmed by reference standard** | **PPV (95% CI)** | **N events identified** | **N events confirmed by reference standard** | **PPV (95% CI)** | **N events identified** | **N events confirmed by reference standard** | **PPV (95% CI)** | **N events identified** | **N events confirmed by reference standard** | **PPV (95% CI)** |
| --- | --- | --- | --- | --- | --- | --- | --- | --- | --- | --- | --- | --- |
|  | ≤55 | | | 55 to 64 | | | 65 to 74 | | | ≥ 75 |  |  |
| **1.OCS prescription** | 288 | 205 | 71.2 (65.6 - 76.3) | 424 | 318 | 75.0 (70.6 - 79.1) | 340 | 245 | 72.1 (67.0 - 76.8) | 100 | 73 | 73.0 (63.2 - 81.4) |
| **2.Antibiotic prescription** | 1234 | 691 | 56.0 (53.2 - 58.8) | 2127 | 1341 | 63.0 (61.0 - 65.1) | 1818 | 1130 | 62.2 (59.9 - 64.4) | 661 | 397 | 60.1 (56.2 - 63.8) |
| **3.OCS and antibiotic prescription** | 210 | 168 | 80.0 (73.9 - 85.2) | 300 | 230 | 76.7 (71.5 - 81.3) | 247 | 200 | 81.0 (75.5 - 85.7) | 66 | 55 | 83.3 (72.1 - 91.4) |
| **4. Symptom definition** | 24 | 15 | 62.5 (40.6 - 81.2) | 47 | 31 | 66.0 (50.7 - 79.1) | 45 | 26 | 57.8 (42.2 - 72.3) | 26 | 20 | 76.9 (56.4 - 91.0) |
| **5. Symptom definition and OCS prescription** | 15 | 12 | 80.0 (51.9 - 95.7) | 28 | 26 | 92.9 (76.5 - 99.1) | 26 | 24 | 92.3 (74.9 - 99.1) | 19 | 17 | 89.5 (66.9 - 98.7) |
| **6. Symptom definition and antibiotic prescription** | 8 | 8 | 100.0 (63.1 - 100.0) | 19 | 17 | 89.5 (66.9 - 98.7) | 17 | 15 | 88.2 (63.6 - 98.5) | 13 | 13 | 100.0 (75.3 - 100.0) |
| **7. Symptom definition and OCS & antibiotic prescription** | 7 | 7 | 100.0 (59.0 - 100.0) | 15 | 15 | 100.0 (78.2 - 100.0) | 15 | 14 | 93.3 (68.1 - 99.8) | 11 | 11 | 100.0 (71.5 - 100.0) |
| **8. LRTI code** | 377 | 288 | 76.4 (71.8 - 80.6) | 645 | 501 | 77.7 (74.3 - 80.8) | 530 | 440 | 83.0 (79.5 - 86.1) | 193 | 160 | 82.9 (76.8 - 87.9) |
| **9. LRTI code and OCS prescription** | 336 | 265 | 78.9 (74.1 - 83.1) | 580 | 463 | 79.8 (76.3 - 83.0) | 473 | 402 | 85.0 (81.4 - 88.1) | 169 | 138 | 81.7 (75.0 - 87.2) |
| **10. LRTI code and antibiotic prescription** | 106 | 93 | 87.7 (79.9 - 93.3) | 129 | 115 | 89.1 (82.5 - 93.9) | 112 | 97 | 86.6 (78.9 - 92.3) | 46 | 42 | 91.3 (79.2 - 97.6) |
| **11. LRTI code and OCS & antibiotic prescription** | 97 | 86 | 88.7 (80.6 - 94.2) | 121 | 107 | 88.4 (81.3 - 93.5) | 108 | 93 | 86.1 (78.1 - 92.0) | 45 | 41 | 91.1 (78.8 - 97.5) |
| **12. AECOPD code** | 135 | 129 | 95.6 (90.6 - 98.4) | 385 | 372 | 96.6 (94.3 - 98.2) | 281 | 269 | 95.7 (92.7 - 97.8) | 84 | 80 | 95.2 (88.3 - 98.7) |
| **13. AECOPD code and OCS prescription** | 95 | 92 | 96.8 (91.0 - 99.3) | 289 | 281 | 97.2 (94.6 - 98.8) | 200 | 193 | 96.5 (92.9 - 98.6) | 54 | 52 | 96.3 (87.3 - 99.5) |
| **14. AECOPD code and antibiotic prescription** | 78 | 75 | 96.2 (89.2 - 99.2) | 196 | 190 | 96.9 (93.5 - 98.9) | 114 | 109 | 95.6 (90.1 - 98.6) | 35 | 34 | 97.1 (85.1 - 99.9) |
| **15. AECOPD code and OCS & antibiotic prescription** | 70 | 68 | 97.1 (90.1 - 99.7) | 176 | 170 | 96.6 (92.7 - 98.7) | 101 | 98 | 97.0 (91.6 - 99.4) | 30 | 29 | 96.7 (82.8 - 99.9) |
