# Supplementary material for: Validation of the Recording of Acute Exacerbations of COPD in UK Primary Care Electronic Healthcare Records
Source: PLoS One. 2016 Mar 9;11(3):e0151357. doi: 10.1371/journal.pone.0151357 (PMC4784784; doi:10.1371/journal.pone.0151357)
Supplement: S2 File — (DOCX) [file pone.0151357.s002.docx]

**CODES USED TO CONSTRUCT AECOPD ALGORITHMS**

**Lower respiratory tract infection diagnostic codes**

| **Medical code** | **Read term** |
| --- | --- |
| 68 | Chest infection |
| 312 | Acute bronchitis |
| 556 | Influenza |
| 1019 | Acute bronchiolitis |
| 1382 | Acute viral bronchitis unspecified |
| 2157 | Flu like illness |
| 2476 | Chest cold |
| 2581 | Chest infection NOS |
| 3358 | Lower resp tract infection |
| 5947 | Influenza like illness |
| 5978 | Acute wheezy bronchitis |
| 6124 | Acute lower respiratory tract infection |
| 6181 | Obliterating fibrous bronchiolitis |
| 8980 | Influenza-like symptoms |
| 9043 | Acute pneumococcal bronchitis |
| 11072 | Acute purulent bronchitis |
| 14791 | Influenza with gastrointestinal tract involvement |
| 15774 | Influenza with laryngitis |
| 16388 | Influenza NOS |
| 17185 | Acute bronchiolitis with bronchospasm |
| 17359 | Chest infection - unspecified bronchitis |
| 17917 | Acute bronchiolitis NOS |
| 18451 | Acute bronchiolitis due to respiratory syncytial virus |
| 20198 | Acute bronchitis NOS |
| 21061 | Chronic obstruct pulmonary dis with acute lower resp infectn |
| 21145 | Acute croupous bronchitis |
| 21492 | Acute haemophilus influenzae bronchitis |
| 23488 | Influenza with respiratory manifestations NOS |
| 24316 | Chest infection with infectious disease EC |
| 24800 | Acute bacterial bronchitis unspecified |
| 26125 | Bronchiolitis obliterans |
| 29273 | Acute bronchitis due to parainfluenza virus |
| 29617 | Influenza with pharyngitis |
| 29669 | Acute bronchitis and bronchiolitis |
| 31363 | Influenza with other manifestations NOS |
| 37447 | Acute lower respiratory tract infection |
| 41137 | Acute bronchitis or bronchiolitis NOS |
| 41589 | Acute obliterating bronchiolitis |
| 43362 | Acute streptococcal bronchitis |
| 43625 | Influenza with other respiratory manifestation |
| 46157 | Influenza with encephalopathy |
| 47472 | Influenza with other manifestations |
| 48593 | Acute bronchitis due to respiratory syncytial virus |
| 49794 | Acute neisseria catarrhalis bronchitis |
| 54533 | Acute capillary bronchiolitis |
| 63697 | Avian influenza virus nucleic acid detection |
| 64890 | Acute bronchitis due to rhinovirus |
| 65916 | Acute bronchitis due to echovirus |
| 66228 | Acute bronchiolitis due to other specified organisms |
| 66397 | [X]Other acute lower respiratory infections |
| 69192 | Acute exudative bronchiolitis |
| 71370 | Acute pseudomembranous bronchitis |
| 73100 | [X]Acute bronchitis due to other specified organisms |
| 91123 | Parainfluenza type 3 nucleic acid detection |
| 93153 | Acute bronchitis due to coxsackievirus |
| 94130 | Parainfluenza type 1 nucleic acid detection |
| 94858 | Parainfluenza type 2 nucleic acid detection |
| 94930 | Avian influenza |
| 96017 | Influenza B virus detected |
| 96018 | Influenza H3 virus detected |
| 96019 | Influenza H1 virus detected |
| 96286 | Human parainfluenza virus detected |
| 97062 | Influenza A virus, other or untyped strain detected |
| 97279 | [X]Influenza+other manifestations, virus not identified |
| 97605 | [X]Influenza+oth respiratory manifestatns,virus not identifd |
| 97936 | [X]Influenza+other manifestations,influenza virus identified |
| 98102 | Influenza A (H1N1) swine flu |
| 98103 | Possible influenza A virus H1N1 subtype |
| 98115 | Suspected swine influenza |
| 98125 | Suspected influenza A virus subtype H1N1 infection |
| 98129 | Influenza due to Influenza A virus subtype H1N1 |
| 98143 | Influenza A virus H1N1 subtype detected |
| 98156 | Influenza H5 virus detected |
| 98257 | [X]Flu+oth respiratory manifestations,'flu virus identified |
| 99214 | [X]Acute bronchiolitis due to other specified organisms |
| 101775 | Acute membranous bronchitis |
| 102918 | Influenza H2 virus detected |

**Acute exacerbation of COPD diagnostic codes**

| **Medical code** | **Read term** |
| --- | --- |
| 1446 | Acute exacerbation of chronic obstructive airways disease |
| 7884 | Chron obstruct pulmonary dis wth acute exacerbation, unspec |

**Cough codes**

| **Medical code** | **Read term** |
| --- | --- |
| 92 | Cough |
| 292 | Chesty cough |
| 1025 | Bronchial cough |
| 1160 | [D]Cough |
| 1234 | Productive cough NOS |
| 1273 | C/O - cough |
| 3068 | Night cough present |
| 3645 | Coughing up phlegm |
| 4070 | Morning cough |
| 4836 | Nocturnal cough / wheeze |
| 4931 | Dry cough |
| 7706 | Productive cough -clear sputum |
| 7707 | Cough symptom NOS |
| 7708 | Productive cough-yellow sputum |
| 7773 | Productive cough -green sputum |
| 8239 | [D]Cough with haemorrhage |
| 18907 | Cough with fever |
| 22318 | Difficulty in coughing up sputum |
| 29318 | Evening cough |
| 60903 | Cough aggravates symptom |
| 100515 | Cough swab |

**Breathlessness codes**

| **Medical code** | **Read term** |
| --- | --- |
| 735 | [D]Breathlessness |
| 741 | [D]Shortness of breath |
| 1429 | Breathlessness |
| 2563 | [D]Respiratory distress |
| 2575 | Short of breath on exertion |
| 2737 | Respiratory distress syndrome |
| 2931 | Difficulty breathing |
| 3092 | [D]Dyspnoea |
| 4822 | Shortness of breath |
| 5175 | Breathlessness symptom |
| 5349 | Shortness of breath symptom |
| 5896 | Dyspnoea - symptom |
| 6326 | Breathless - moderate exertion |
| 6434 | Paroxysmal nocturnal dyspnoea |
| 7000 | O/E - dyspnoea |
| 7534 | O/E - respiratory distress |
| 7683 | Breathless - lying flat |
| 7932 | Breathless - mild exertion |
| 9297 | [D]Respiratory insufficiency |
| 18116 | Nocturnal dyspnoea |
| 21801 | Breathlessness NOS |
| 22094 | Short of breath dressing/undressing |
| 24889 | Breathless - strenuous exertion |
| 31143 | Breathless - at rest |
| 40813 | Unable to complete a sentence in one breath |
| 53771 | Dyspnoea on exertion |

**Sputum codes**

| **Medical codes** | **Read term** |
| --- | --- |
| 292 | Chesty cough |
| 1025 | Bronchial cough |
| 1234 | Productive cough NOS |
| 1251 | [D]Abnormal sputum |
| 3645 | Coughing up phlegm |
| 3727 | Sputum sent for C/S |
| 7706 | Productive cough -clear sputum |
| 7708 | Productive cough-yellow sputum |
| 7773 | Productive cough -green sputum |
| 8287 | Sputum sample obtained |
| 8760 | [D]Positive culture findings in sputum |
| 9807 | Sputum - symptom |
| 11072 | Acute purulent bronchitis |
| 14271 | Sputum culture |
| 14272 | Sputum microscopy |
| 14273 | Sputum appearance |
| 14804 | Sputum appears infected |
| 15430 | [D]Sputum abnormal - colour |
| 16026 | Sputum examination: abnormal |
| 18964 | Sputum clearance |
| 20086 | [D]Sputum abnormal - amount |
| 22318 | Difficulty in coughing up sputum |
| 23252 | Sputum microscopy NOS |
| 23582 | [D]Abnormal sputum NOS |
| 24181 | Sputum: mucopurulent |
| 30754 | Yellow sputum |
| 30904 | Sputum sent for examination |
| 36515 | [D]Abnormal sputum - tenacious |
| 36880 | Green sputum |
| 43270 | Sputum evidence of infection |
| 44214 | [D]Sputum abnormal - odour |
| 49144 | Sputum: pus cells present |
| 49694 | Sputum: organism on gram stain |
| 54177 | Sputum: excessive - mucoid |
| 100484 | Volume of sputum |
| 100524 | Moderate sputum |
| 100629 | White sputum |
| 100647 | Copious sputum |
| 100931 | Brown sputum |
| 101782 | Profuse sputum |
| 103209 | Grey sputum |

**COPD specific oral corticosteroid codes**

| **Product code** | **Product name** |
| --- | --- |
| 95 | prednisolone 5mg tablets |
| 1063 | prednesol 5mg tablet (sovereign medical ltd) |
| 2044 | prednisone 2.5 mg tab |
| 2368 | prednisolone 2.5mg tablet |
| 2390 | prednisolone e/c 1 mg tab |
| 2799 | prednisolone 10 mg tab |
| 2949 | prednisone 5mg tablets |
| 3059 | prednisolone 50 mg tab |
| 3345 | sintisone tablet (pharmacia ltd) |
| 3557 | prednisone 1mg tablets |
| 7584 | prednisolone 4 mg tab |
| 7710 | prednisolone 15 mg tab |
| 7934 | prednisone 30 mg tab |
| 9727 | prednisolone 50mg tablets |
| 13522 | prednisolone 2 mg tab |
| 13615 | prednisone 10 mg tab |
| 16724 | prednisone 50 mg tab |
| 20095 | precortisyl forte 25mg tablet (aventis pharma) |
| 20670 | prednisolone e/c |
| 21833 | decortisyl 5mg tablet (roussel laboratories ltd) |
| 23512 | precortisyl 5mg tablet (hoechst marion roussel) |
| 24716 | prednisolone e/c |
| 25272 | precortisyl 1mg tablet (hoechst marion roussel) |
| 27889 | prednisolone |
| 27959 | prednisolone |
| 27962 | deltastab 1mg tablet (waymade healthcare plc) |
| 28376 | prednisolone 2.5mg gastro-resistant tablet (biorex laboratories ltd) |
| 28859 | deltastab 5mg tablet (waymade healthcare plc) |
| 30390 | deltastab 2 mg tab |
| 30971 | decortisyl 25 mg tab |
| 31327 | prednisolone steaglate 6.65mg tablet |
| 33691 | prednisolone 5mg gastro-resistant tablet (biorex laboratories ltd) |
| 33988 | prednisolone 5mg tablet (co-pharma ltd) |
| 33990 | prednisolone 5mg tablet (ivax pharmaceuticals uk ltd) |
| 34109 | prednisolone 5 mg gastro-resistant tablet |
| 34631 | prednisolone 1mg tablet (co-pharma ltd) |
| 34914 | prednisolone 1mg tablet (celltech pharma europe ltd) |
| 38407 | prednisolone 20mg tablet |
| 43544 | prednisone 5mg tablet (knoll ltd) |
| 44380 | prednisone 1mg modified-release tablets |
| 44723 | prednisone 5mg modified-release tablets |
| 44802 | lodotra 5mg modified-release tablets (napp pharmaceuticals ltd) |
| 44803 | lodotra 2mg modified-release tablets (napp pharmaceuticals ltd) |
| 45302 | prednisolone 5mg tablet (biorex laboratories ltd) |
| 46711 | prednisone 2mg modified-release tablets |
| 47142 | prednisolone 5mg soluble tablet (amdipharm plc) |
| 54432 | lodotra 1mg modified-release tablets (napp pharmaceuticals ltd) |

**COPD specific antibiotic codes**

| **Product code** | **Product name** |
| --- | --- |
| 22029 | amiclav 250mg/125mg tablets (ashbourne pharmaceuticals ltd) |
| 11634 | amix 125 oral suspension (ashbourne pharmaceuticals ltd) |
| 11613 | amix 250 capsules (ashbourne pharmaceuticals ltd) |
| 21844 | amix 250 oral suspension (ashbourne pharmaceuticals ltd) |
| 18786 | amix 500 capsules (ashbourne pharmaceuticals ltd) |
| 29697 | amopen 125mg/5ml liquid (yorkshire pharmaceuticals ltd) |
| 30498 | amopen 250mg capsule (yorkshire pharmaceuticals ltd) |
| 31423 | amopen 250mg/5ml liquid (yorkshire pharmaceuticals ltd) |
| 17711 | amopen 500mg capsule (yorkshire pharmaceuticals ltd) |
| 12378 | amoram 125mg/5ml oral suspension (lpc medical (uk) ltd) |
| 9243 | amoram 250mg capsules (lpc medical (uk) ltd) |
| 22438 | amoram 250mg/5ml oral suspension (lpc medical (uk) ltd) |
| 22415 | amoram 500mg capsules (lpc medical (uk) ltd) |
| 8906 | amoxicillin 125mg / clavulanic acid 31mg/5ml oral suspension |
| 13285 | amoxicillin 125mg / clavulanic acid 31mg/5ml oral suspension |
| 53942 | amoxicillin 125mg / clavulanic acid 62.5mg/5ml oral suspension |
| 41835 | amoxicillin 125mg powder (ivax pharmaceuticals uk ltd) |
| 3742 | amoxicillin 125mg sugar free chewable tablets |
| 13848 | amoxicillin 125mg sugar free powder |
| 485 | amoxicillin 125mg/1.25ml oral suspension paediatric |
| 42822 | amoxicillin 125mg/5ml mixture (celltech pharma europe ltd) |
| 28872 | amoxicillin 125mg/5ml mixture (crosspharma ltd) |
| 41818 | amoxicillin 125mg/5ml oral solution (berk pharmaceuticals ltd) |
| 42240 | amoxicillin 125mg/5ml oral solution (co-pharma ltd) |
| 29337 | amoxicillin 125mg/5ml oral solution (neo laboratories ltd) |
| 62 | amoxicillin 125mg/5ml oral suspension |
| 33690 | amoxicillin 125mg/5ml oral suspension (a a h pharmaceuticals ltd) |
| 34857 | amoxicillin 125mg/5ml oral suspension (actavis uk ltd) |
| 42545 | amoxicillin 125mg/5ml oral suspension (almus pharmaceuticals ltd) |
| 50002 | amoxicillin 125mg/5ml oral suspension (bristol laboratories ltd) |
| 32622 | amoxicillin 125mg/5ml oral suspension (generics (uk) ltd) |
| 23238 | amoxicillin 125mg/5ml oral suspension (ivax pharmaceuticals uk ltd) |
| 48038 | amoxicillin 125mg/5ml oral suspension (kent pharmaceuticals ltd) |
| 52685 | amoxicillin 125mg/5ml oral suspension (phoenix healthcare distribution ltd) |
| 28875 | amoxicillin 125mg/5ml oral suspension (ranbaxy (uk) ltd) |
| 43229 | amoxicillin 125mg/5ml oral suspension (sandoz ltd) |
| 55047 | amoxicillin 125mg/5ml oral suspension (sandoz ltd) |
| 28870 | amoxicillin 125mg/5ml oral suspension (teva uk ltd) |
| 56561 | amoxicillin 125mg/5ml oral suspension (waymade healthcare plc) |
| 503 | amoxicillin 125mg/5ml oral suspension sugar free |
| 33696 | amoxicillin 125mg/5ml oral suspension sugar free (a a h pharmaceuticals ltd) |
| 34679 | amoxicillin 125mg/5ml oral suspension sugar free (actavis uk ltd) |
| 53078 | amoxicillin 125mg/5ml oral suspension sugar free (alliance healthcare (distribution) ltd) |
| 36054 | amoxicillin 125mg/5ml oral suspension sugar free (almus pharmaceuticals ltd) |
| 52122 | amoxicillin 125mg/5ml oral suspension sugar free (bristol laboratories ltd) |
| 31014 | amoxicillin 125mg/5ml oral suspension sugar free (generics (uk) ltd) |
| 24150 | amoxicillin 125mg/5ml oral suspension sugar free (ivax pharmaceuticals uk ltd) |
| 34384 | amoxicillin 125mg/5ml oral suspension sugar free (kent pharmaceuticals ltd) |
| 52857 | amoxicillin 125mg/5ml oral suspension sugar free (phoenix healthcare distribution ltd) |
| 29858 | amoxicillin 125mg/5ml oral suspension sugar free (sandoz ltd) |
| 34638 | amoxicillin 125mg/5ml oral suspension sugar free (teva uk ltd) |
| 55626 | amoxicillin 125mg/5ml oral suspension sugar free (waymade healthcare plc) |
| 1391 | amoxicillin 250mg / clavulanic acid 125mg tablets |
| 7636 | amoxicillin 250mg / clavulanic acid 62mg/5ml oral suspension |
| 13262 | amoxicillin 250mg / clavulanic acid 62mg/5ml oral suspension |
| 42809 | amoxicillin 250mg capsule (c p pharmaceuticals ltd) |
| 31661 | amoxicillin 250mg capsule (co-pharma ltd) |
| 28882 | amoxicillin 250mg capsule (crosspharma ltd) |
| 34435 | amoxicillin 250mg capsule (ddsa pharmaceuticals ltd) |
| 33222 | amoxicillin 250mg capsule (lagap) |
| 32872 | amoxicillin 250mg capsule (mepra-pharm) |
| 34714 | amoxicillin 250mg capsule (neo laboratories ltd) |
| 45267 | amoxicillin 250mg capsule (regent laboratories ltd) |
| 9 | amoxicillin 250mg capsules |
| 25484 | amoxicillin 250mg capsules (a a h pharmaceuticals ltd) |
| 33343 | amoxicillin 250mg capsules (actavis uk ltd) |
| 54796 | amoxicillin 250mg capsules (boston healthcare ltd) |
| 54491 | amoxicillin 250mg capsules (bristol laboratories ltd) |
| 30745 | amoxicillin 250mg capsules (generics (uk) ltd) |
| 34042 | amoxicillin 250mg capsules (ivax pharmaceuticals uk ltd) |
| 30528 | amoxicillin 250mg capsules (kent pharmaceuticals ltd) |
| 54271 | amoxicillin 250mg capsules (mawdsley-brooks & company ltd) |
| 51536 | amoxicillin 250mg capsules (milpharm ltd) |
| 30743 | amoxicillin 250mg capsules (ranbaxy (uk) ltd) |
| 48006 | amoxicillin 250mg capsules (sandoz ltd) |
| 23967 | amoxicillin 250mg capsules (teva uk ltd) |
| 54185 | amoxicillin 250mg capsules (wockhardt uk ltd) |
| 870 | amoxicillin 250mg sugar free chewable tablets |
| 42815 | amoxicillin 250mg/5ml mixture (celltech pharma europe ltd) |
| 33570 | amoxicillin 250mg/5ml mixture (crosspharma ltd) |
| 40238 | amoxicillin 250mg/5ml mixture (mepra-pharm) |
| 45317 | amoxicillin 250mg/5ml oral solution (neo laboratories ltd) |
| 427 | amoxicillin 250mg/5ml oral suspension |
| 33165 | amoxicillin 250mg/5ml oral suspension (a a h pharmaceuticals ltd) |
| 34760 | amoxicillin 250mg/5ml oral suspension (actavis uk ltd) |
| 41090 | amoxicillin 250mg/5ml oral suspension (almus pharmaceuticals ltd) |
| 55018 | amoxicillin 250mg/5ml oral suspension (bristol laboratories ltd) |
| 33689 | amoxicillin 250mg/5ml oral suspension (generics (uk) ltd) |
| 32640 | amoxicillin 250mg/5ml oral suspension (ivax pharmaceuticals uk ltd) |
| 51382 | amoxicillin 250mg/5ml oral suspension (phoenix healthcare distribution ltd) |
| 55499 | amoxicillin 250mg/5ml oral suspension (ranbaxy (uk) ltd) |
| 56223 | amoxicillin 250mg/5ml oral suspension (sandoz ltd) |
| 37755 | amoxicillin 250mg/5ml oral suspension (sandoz ltd) |
| 53924 | amoxicillin 250mg/5ml oral suspension (sigma pharmaceuticals plc) |
| 27725 | amoxicillin 250mg/5ml oral suspension (teva uk ltd) |
| 585 | amoxicillin 250mg/5ml oral suspension sugar free |
| 34232 | amoxicillin 250mg/5ml oral suspension sugar free (a a h pharmaceuticals ltd) |
| 40243 | amoxicillin 250mg/5ml oral suspension sugar free (actavis uk ltd) |
| 54222 | amoxicillin 250mg/5ml oral suspension sugar free (alliance healthcare (distribution) ltd) |
| 42732 | amoxicillin 250mg/5ml oral suspension sugar free (almus pharmaceuticals ltd) |
| 49065 | amoxicillin 250mg/5ml oral suspension sugar free (bristol laboratories ltd) |
| 31535 | amoxicillin 250mg/5ml oral suspension sugar free (generics (uk) ltd) |
| 33699 | amoxicillin 250mg/5ml oral suspension sugar free (ivax pharmaceuticals uk ltd) |
| 34855 | amoxicillin 250mg/5ml oral suspension sugar free (kent pharmaceuticals ltd) |
| 34775 | amoxicillin 250mg/5ml oral suspension sugar free (teva uk ltd) |
| 17746 | amoxicillin 375mg soluble tablets |
| 1140 | amoxicillin 3g oral powder sachets sugar free |
| 33383 | amoxicillin 3g oral powder sachets sugar free (a a h pharmaceuticals ltd) |
| 40168 | amoxicillin 3g oral powder sachets sugar free (kent pharmaceuticals ltd) |
| 28130 | amoxicillin 3g oral powder sachets sugar free (teva uk ltd) |
| 41734 | amoxicillin 3g powder (actavis uk ltd) |
| 15192 | amoxicillin 400mg / clavulanic acid 57mg/5ml sugar free oral suspension |
| 5662 | amoxicillin 500mg / clarithromycin 500mg / lansoprazole 30mg triple pack |
| 13216 | amoxicillin 500mg / clavulanic acid 125mg tablets |
| 38684 | amoxicillin 500mg capsule (c p pharmaceuticals ltd) |
| 35570 | amoxicillin 500mg capsule (crosspharma ltd) |
| 34885 | amoxicillin 500mg capsule (ddsa pharmaceuticals ltd) |
| 44854 | amoxicillin 500mg capsule (lagap) |
| 34912 | amoxicillin 500mg capsule (neo laboratories ltd) |
| 48 | amoxicillin 500mg capsules |
| 33692 | amoxicillin 500mg capsules (a a h pharmaceuticals ltd) |
| 53627 | amoxicillin 500mg capsules (accord healthcare ltd) |
| 26157 | amoxicillin 500mg capsules (actavis uk ltd) |
| 52820 | amoxicillin 500mg capsules (alliance healthcare (distribution) ltd) |
| 47640 | amoxicillin 500mg capsules (almus pharmaceuticals ltd) |
| 55527 | amoxicillin 500mg capsules (boston healthcare ltd) |
| 52771 | amoxicillin 500mg capsules (bristol laboratories ltd) |
| 23740 | amoxicillin 500mg capsules (generics (uk) ltd) |
| 29463 | amoxicillin 500mg capsules (ivax pharmaceuticals uk ltd) |
| 33706 | amoxicillin 500mg capsules (kent pharmaceuticals ltd) |
| 52058 | amoxicillin 500mg capsules (medreich plc) |
| 54725 | amoxicillin 500mg capsules (milpharm ltd) |
| 34852 | amoxicillin 500mg capsules (ranbaxy (uk) ltd) |
| 31801 | amoxicillin 500mg capsules (sandoz ltd) |
| 34001 | amoxicillin 500mg capsules (teva uk ltd) |
| 55394 | amoxicillin 500mg capsules (wockhardt uk ltd) |
| 1722 | amoxicillin 500mg dispersible tablets |
| 2281 | amoxicillin 500mg sugar free chewable tablets |
| 4582 | amoxicillin 750mg soluble tablets |
| 9343 | amoxicillin 750mg sugar free powder |
| 439 | amoxicillin with clavulanic acid dispersible tablets |
| 2171 | amoxil 125mg/1.25ml paediatric oral suspension (glaxosmithkline uk ltd) |
| 2153 | amoxil 125mg/5ml syrup sucrose free (glaxosmithkline uk ltd) |
| 133 | amoxil 250mg capsules (glaxosmithkline uk ltd) |
| 1812 | amoxil 250mg/5ml syrup sucrose free (glaxosmithkline uk ltd) |
| 2174 | amoxil 3g oral powder sachets sucrose free (glaxosmithkline uk ltd) |
| 847 | amoxil 500mg capsules (glaxosmithkline uk ltd) |
| 49590 | amoxil 500mg capsules (lexon (uk) ltd) |
| 51436 | amoxil 500mg capsules (mawdsley-brooks & company ltd) |
| 56700 | amoxil 500mg capsules (necessity supplies ltd) |
| 15148 | amoxil 500mg dispersible tablet (smithkline beecham plc) |
| 4010 | amoxil 750mg sachets (glaxosmithkline uk ltd) |
| 4154 | amoxil fiztab 125mg tablet (bencard) |
| 1637 | amoxil fiztab 250mg tablet (bencard) |
| 7737 | amoxil fiztab 500mg tablet (bencard) |
| 31571 | amoxycillin |
| 32505 | amoxycillin |
| 27897 | amoxycillin |
| 7592 | amoxycillin 125 mg cap |
| 22469 | amoxycillin 125mg/31mg clavulanic acid |
| 25034 | amoxycillin 125mg/62mg clavulanic acid |
| 7581 | amoxycillin 125mg/62mg clavulanic acid syr |
| 27886 | amoxycillin 250/clavulanic acid 125 disp |
| 19795 | amoxycillin 250mg/clavulanic acid 125mg |
| 1570 | amoxycillin 500 mg tab |
| 2902 | amoxycillin fiztab 125 mg tab |
| 1393 | amoxycillin fiztab 250 mg tab |
| 22293 | amoxycillin trihydrate sachet |
| 21982 | amoxycillin trihydrate sachet |
| 31286 | amoxymed 125mg/5ml oral solution (medipharma ltd) |
| 3669 | amoxymed 250mg capsule (medipharma ltd) |
| 33109 | amrit 125mg/5ml liquid (bhr pharmaceuticals ltd) |
| 27714 | amrit 250mg capsule (bhr pharmaceuticals ltd) |
| 33110 | amrit 250mg/5ml liquid (bhr pharmaceuticals ltd) |
| 33112 | amrit 500mg capsule (bhr pharmaceuticals ltd) |
| 27495 | arpimycin 125mg/5ml liquid (rosemont pharmaceuticals ltd) |
| 36544 | arpimycin 125mg/5ml oral suspension (rosemont pharmaceuticals ltd) |
| 24220 | arpimycin 250mg/5ml liquid (rosemont pharmaceuticals ltd) |
| 36514 | arpimycin 250mg/5ml oral suspension (rosemont pharmaceuticals ltd) |
| 37022 | arpimycin 500mg/5ml liquid (rosemont pharmaceuticals ltd) |
| 415 | augmentin 125/31 sf oral suspension (glaxosmithkline uk ltd) |
| 50595 | augmentin 125/31 sf oral suspension (mawdsley-brooks & company ltd) |
| 51164 | augmentin 125/31 sf oral suspension (waymade healthcare plc) |
| 569 | augmentin 250/62 sf oral suspension (glaxosmithkline uk ltd) |
| 52666 | augmentin 250/62 sf oral suspension (sigma pharmaceuticals plc) |
| 2507 | augmentin 375mg dispersible tablets (glaxosmithkline uk ltd) |
| 49063 | augmentin 375mg tablets (doncaster pharmaceuticals ltd) |
| 399 | augmentin 375mg tablets (glaxosmithkline uk ltd) |
| 48683 | augmentin 375mg tablets (lexon (uk) ltd) |
| 49374 | augmentin 375mg tablets (mawdsley-brooks & company ltd) |
| 49048 | augmentin 375mg tablets (waymade healthcare plc) |
| 50279 | augmentin 625mg tablets (doncaster pharmaceuticals ltd) |
| 509 | augmentin 625mg tablets (glaxosmithkline uk ltd) |
| 49656 | augmentin 625mg tablets (lexon (uk) ltd) |
| 52207 | augmentin 625mg tablets (mawdsley-brooks & company ltd) |
| 49321 | augmentin 625mg tablets (sigma pharmaceuticals plc) |
| 49683 | augmentin 625mg tablets (waymade healthcare plc) |
| 5341 | augmentin-duo 400/57 oral suspension (glaxosmithkline uk ltd) |
| 56591 | augmentin-duo 400/57 oral suspension (lexon (uk) ltd) |
| 51194 | augmentin-duo 400/57 oral suspension (sigma pharmaceuticals plc) |
| 31007 | aureomycin powder (wyeth pharmaceuticals) |
| 25127 | avelox 400mg tablets (bayer plc) |
| 26289 | bacticlor mr 375mg tablets (ranbaxy (uk) ltd) |
| 4895 | benzoyl peroxide 5% / erythromycin 3% gel |
| 21802 | berkmycen 250mg tablet (berk pharmaceuticals ltd) |
| 17093 | bisolvomycin capsule (boehringer ingelheim ltd) |
| 13910 | cefaclor 125mg/5ml liquid (generics (uk) ltd) |
| 14607 | cefaclor 125mg/5ml liquid (lagap) |
| 1038 | cefaclor 125mg/5ml oral suspension |
| 39703 | cefaclor 125mg/5ml oral suspension (a a h pharmaceuticals ltd) |
| 34913 | cefaclor 125mg/5ml oral suspension (genus pharmaceuticals ltd) |
| 32235 | cefaclor 125mg/5ml oral suspension (ranbaxy (uk) ltd) |
| 7526 | cefaclor 125mg/5ml oral suspension sugar free |
| 56610 | cefaclor 125mg/5ml oral suspension sugar free (phoenix healthcare distribution ltd) |
| 9520 | cefaclor 250mg capsule (lagap) |
| 366 | cefaclor 250mg capsules |
| 30772 | cefaclor 250mg capsules (ranbaxy (uk) ltd) |
| 20420 | cefaclor 250mg/5ml liquid (generics (uk) ltd) |
| 20409 | cefaclor 250mg/5ml liquid (lagap) |
| 3737 | cefaclor 250mg/5ml oral suspension |
| 46973 | cefaclor 250mg/5ml oral suspension (genus pharmaceuticals ltd) |
| 48025 | cefaclor 250mg/5ml oral suspension (ranbaxy (uk) ltd) |
| 9293 | cefaclor 250mg/5ml oral suspension sugar free |
| 3180 | cefaclor 375mg modified-release tablets |
| 34838 | cefaclor 375mg modified-release tablets (a a h pharmaceuticals ltd) |
| 20881 | cefaclor 375mg modified-release tablets (ranbaxy (uk) ltd) |
| 4689 | cefaclor 500mg capsule (lagap) |
| 2976 | cefaclor 500mg capsules |
| 43425 | cefaclor 500mg capsules (a a h pharmaceuticals ltd) |
| 55211 | cefaclor 500mg capsules (kent pharmaceuticals ltd) |
| 30771 | cefaclor 500mg capsules (ranbaxy (uk) ltd) |
| 8051 | cefaclor 500mg modified-release tablets |
| 12248 | cefalexin 125mg/1.25ml paediatric drops |
| 1693 | cefalexin 125mg/5ml oral suspension |
| 29748 | cefalexin 125mg/5ml oral suspension (a a h pharmaceuticals ltd) |
| 32181 | cefalexin 125mg/5ml oral suspension (actavis uk ltd) |
| 53945 | cefalexin 125mg/5ml oral suspension (alliance healthcare (distribution) ltd) |
| 39417 | cefalexin 125mg/5ml oral suspension (generics (uk) ltd) |
| 32642 | cefalexin 125mg/5ml oral suspension (kent pharmaceuticals ltd) |
| 36578 | cefalexin 125mg/5ml oral suspension (ranbaxy (uk) ltd) |
| 33329 | cefalexin 125mg/5ml oral suspension (teva uk ltd) |
| 6651 | cefalexin 125mg/5ml oral suspension sugar free |
| 19144 | cefalexin 125mg/5ml oral suspension sugar free (teva uk ltd) |
| 1384 | cefalexin 125mg/5ml suspension |
| 18451 | cefalexin 1g tablets |
| 33802 | cefalexin 250mg capsule (berk pharmaceuticals ltd) |
| 155 | cefalexin 250mg capsules |
| 34253 | cefalexin 250mg capsules (a a h pharmaceuticals ltd) |
| 19152 | cefalexin 250mg capsules (actavis uk ltd) |
| 54864 | cefalexin 250mg capsules (alliance healthcare (distribution) ltd) |
| 52283 | cefalexin 250mg capsules (arrow generics ltd) |
| 19160 | cefalexin 250mg capsules (generics (uk) ltd) |
| 19133 | cefalexin 250mg capsules (ivax pharmaceuticals uk ltd) |
| 41736 | cefalexin 250mg capsules (kent pharmaceuticals ltd) |
| 52282 | cefalexin 250mg capsules (milpharm ltd) |
| 24090 | cefalexin 250mg capsules (pliva pharma ltd) |
| 36599 | cefalexin 250mg capsules (ranbaxy (uk) ltd) |
| 9690 | cefalexin 250mg capsules (teva uk ltd) |
| 40747 | cefalexin 250mg chewable tablets |
| 1146 | cefalexin 250mg tablets |
| 33334 | cefalexin 250mg tablets (a a h pharmaceuticals ltd) |
| 36330 | cefalexin 250mg tablets (actavis uk ltd) |
| 47163 | cefalexin 250mg tablets (arrow generics ltd) |
| 36701 | cefalexin 250mg tablets (generics (uk) ltd) |
| 31825 | cefalexin 250mg tablets (ivax pharmaceuticals uk ltd) |
| 9698 | cefalexin 250mg tablets (teva uk ltd) |
| 41825 | cefalexin 250mg/5ml oral solution (c p pharmaceuticals ltd) |
| 1860 | cefalexin 250mg/5ml oral suspension |
| 42008 | cefalexin 250mg/5ml oral suspension (a a h pharmaceuticals ltd) |
| 45221 | cefalexin 250mg/5ml oral suspension (actavis uk ltd) |
| 29464 | cefalexin 250mg/5ml oral suspension (generics (uk) ltd) |
| 41192 | cefalexin 250mg/5ml oral suspension (ranbaxy (uk) ltd) |
| 41968 | cefalexin 250mg/5ml oral suspension (teva uk ltd) |
| 6671 | cefalexin 250mg/5ml oral suspension sugar free |
| 34133 | cefalexin 250mg/5ml oral suspension sugar free (teva uk ltd) |
| 1713 | cefalexin 250mg/5ml suspension |
| 44755 | cefalexin 500mg capsule (berk pharmaceuticals ltd) |
| 400 | cefalexin 500mg capsules |
| 32643 | cefalexin 500mg capsules (a a h pharmaceuticals ltd) |
| 19138 | cefalexin 500mg capsules (actavis uk ltd) |
| 52851 | cefalexin 500mg capsules (alliance healthcare (distribution) ltd) |
| 19184 | cefalexin 500mg capsules (generics (uk) ltd) |
| 9664 | cefalexin 500mg capsules (ivax pharmaceuticals uk ltd) |
| 36569 | cefalexin 500mg capsules (kent pharmaceuticals ltd) |
| 54955 | cefalexin 500mg capsules (milpharm ltd) |
| 19161 | cefalexin 500mg capsules (ranbaxy (uk) ltd) |
| 29281 | cefalexin 500mg capsules (teva uk ltd) |
| 865 | cefalexin 500mg tablets |
| 29202 | cefalexin 500mg tablets (a a h pharmaceuticals ltd) |
| 22321 | cefalexin 500mg tablets (generics (uk) ltd) |
| 31827 | cefalexin 500mg tablets (ivax pharmaceuticals uk ltd) |
| 9689 | cefalexin 500mg tablets (teva uk ltd) |
| 2227 | cefalexin 500mg/5ml oral suspension |
| 17150 | ceporex 125mg/1.25ml drops (glaxo laboratories ltd) |
| 7560 | ceporex 125mg/5ml liquid (galen ltd) |
| 3609 | ceporex 125mg/5ml oral solution (galen ltd) |
| 41106 | ceporex 125mg/5ml syrup (co-pharma ltd) |
| 12235 | ceporex 1g tablet (galen ltd) |
| 192 | ceporex 250mg capsule (galen ltd) |
| 40884 | ceporex 250mg capsules (co-pharma ltd) |
| 8019 | ceporex 250mg tablet (galen ltd) |
| 41049 | ceporex 250mg tablets (co-pharma ltd) |
| 8625 | ceporex 250mg/5ml liquid (galen ltd) |
| 8008 | ceporex 250mg/5ml oral solution (galen ltd) |
| 40945 | ceporex 250mg/5ml syrup (co-pharma ltd) |
| 2661 | ceporex 500mg capsule (galen ltd) |
| 40915 | ceporex 500mg capsules (co-pharma ltd) |
| 8085 | ceporex 500mg tablet (galen ltd) |
| 40914 | ceporex 500mg tablets (co-pharma ltd) |
| 5859 | ceporex 500mg/5ml oral solution (galen ltd) |
| 41230 | ceporex 500mg/5ml syrup (co-pharma ltd) |
| 7881 | chlortetracycline 250mg capsules |
| 36689 | chlortetracycline hcl syr |
| 12016 | chymocyclar capsule (rorer pharmaceuticals ltd) |
| 27016 | ciprofloxacin |
| 498 | ciprofloxacin 100mg tablets |
| 42507 | ciprofloxacin 100mg tablets (a a h pharmaceuticals ltd) |
| 48031 | ciprofloxacin 100mg tablets (almus pharmaceuticals ltd) |
| 54555 | ciprofloxacin 100mg tablets (doncaster pharmaceuticals ltd) |
| 54674 | ciprofloxacin 100mg tablets (phoenix healthcare distribution ltd) |
| 39913 | ciprofloxacin 100mg tablets (sandoz ltd) |
| 52309 | ciprofloxacin 100mg tablets (sigma pharmaceuticals plc) |
| 52945 | ciprofloxacin 200mg/100ml solution for infusion vials |
| 56439 | ciprofloxacin 200mg/100ml solution for infusion vials (a a h pharmaceuticals ltd) |
| 34647 | ciprofloxacin 250mg tablet (neo laboratories ltd) |
| 281 | ciprofloxacin 250mg tablets |
| 29343 | ciprofloxacin 250mg tablets (a a h pharmaceuticals ltd) |
| 50601 | ciprofloxacin 250mg tablets (accord healthcare ltd) |
| 34308 | ciprofloxacin 250mg tablets (actavis uk ltd) |
| 51537 | ciprofloxacin 250mg tablets (alliance healthcare (distribution) ltd) |
| 54393 | ciprofloxacin 250mg tablets (arrow generics ltd) |
| 54701 | ciprofloxacin 250mg tablets (bristol laboratories ltd) |
| 56381 | ciprofloxacin 250mg tablets (co-pharma ltd) |
| 43814 | ciprofloxacin 250mg tablets (dr reddy's laboratories (uk) ltd) |
| 33989 | ciprofloxacin 250mg tablets (generics (uk) ltd) |
| 41561 | ciprofloxacin 250mg tablets (ivax pharmaceuticals uk ltd) |
| 54302 | ciprofloxacin 250mg tablets (medreich plc) |
| 34448 | ciprofloxacin 250mg tablets (niche generics ltd) |
| 34694 | ciprofloxacin 250mg tablets (pliva pharma ltd) |
| 34559 | ciprofloxacin 250mg tablets (sandoz ltd) |
| 34478 | ciprofloxacin 250mg tablets (teva uk ltd) |
| 34655 | ciprofloxacin 250mg tablets (wockhardt uk ltd) |
| 4091 | ciprofloxacin 250mg/5ml oral suspension |
| 10304 | ciprofloxacin 2mg/ml infusion |
| 45341 | ciprofloxacin 500mg tablet (neo laboratories ltd) |
| 34322 | ciprofloxacin 500mg tablet (niche generics ltd) |
| 583 | ciprofloxacin 500mg tablets |
| 29458 | ciprofloxacin 500mg tablets (a a h pharmaceuticals ltd) |
| 52501 | ciprofloxacin 500mg tablets (accord healthcare ltd) |
| 34605 | ciprofloxacin 500mg tablets (actavis uk ltd) |
| 49445 | ciprofloxacin 500mg tablets (almus pharmaceuticals ltd) |
| 56789 | ciprofloxacin 500mg tablets (apc pharmaceuticals & chemicals (europe) ltd) |
| 52616 | ciprofloxacin 500mg tablets (arrow generics ltd) |
| 53641 | ciprofloxacin 500mg tablets (co-pharma ltd) |
| 50055 | ciprofloxacin 500mg tablets (doncaster pharmaceuticals ltd) |
| 53088 | ciprofloxacin 500mg tablets (dr reddy's laboratories (uk) ltd) |
| 30707 | ciprofloxacin 500mg tablets (generics (uk) ltd) |
| 42174 | ciprofloxacin 500mg tablets (ivax pharmaceuticals uk ltd) |
| 55917 | ciprofloxacin 500mg tablets (medreich plc) |
| 43557 | ciprofloxacin 500mg tablets (pliva pharma ltd) |
| 53878 | ciprofloxacin 500mg tablets (ranbaxy (uk) ltd) |
| 43797 | ciprofloxacin 500mg tablets (sandoz ltd) |
| 45285 | ciprofloxacin 500mg tablets (teva uk ltd) |
| 34494 | ciprofloxacin 500mg tablets (wockhardt uk ltd) |
| 34973 | ciprofloxacin 750mg tablet (niche generics ltd) |
| 1837 | ciprofloxacin 750mg tablets |
| 29472 | ciprofloxacin 750mg tablets (a a h pharmaceuticals ltd) |
| 43517 | ciprofloxacin 750mg tablets (actavis uk ltd) |
| 52099 | ciprofloxacin 750mg tablets (bristol laboratories ltd) |
| 56856 | ciprofloxacin 750mg tablets (ranbaxy (uk) ltd) |
| 28544 | ciprofloxaxin 400mg/200ml in glucose 5% infusion |
| 9154 | ciproxin 100mg tablets (bayer plc) |
| 1202 | ciproxin 250mg tablets (bayer plc) |
| 52353 | ciproxin 250mg tablets (doncaster pharmaceuticals ltd) |
| 53519 | ciproxin 250mg tablets (lexon (uk) ltd) |
| 163 | ciproxin 250mg/5ml oral suspension (bayer plc) |
| 728 | ciproxin 500mg tablets (bayer plc) |
| 52807 | ciproxin 500mg tablets (mawdsley-brooks & company ltd) |
| 52177 | ciproxin 500mg tablets (sigma pharmaceuticals plc) |
| 49839 | ciproxin 500mg tablets (waymade healthcare plc) |
| 7752 | ciproxin 750mg tablets (bayer plc) |
| 45591 | clarie xl 500mg tablets (teva uk ltd) |
| 10326 | clarithromycin 125mg granules straws |
| 331 | clarithromycin 125mg/5ml oral suspension |
| 45795 | clarithromycin 125mg/5ml oral suspension (a a h pharmaceuticals ltd) |
| 54903 | clarithromycin 125mg/5ml oral suspension (alliance healthcare (distribution) ltd) |
| 51831 | clarithromycin 125mg/5ml oral suspension (phoenix healthcare distribution ltd) |
| 41453 | clarithromycin 125mg/5ml oral suspension (ranbaxy (uk) ltd) |
| 53168 | clarithromycin 125mg/5ml oral suspension (sandoz ltd) |
| 26059 | clarithromycin 187.5mg granules straws |
| 765 | clarithromycin 250mg granules sachets |
| 17645 | clarithromycin 250mg granules straws |
| 537 | clarithromycin 250mg tablets |
| 34650 | clarithromycin 250mg tablets (a a h pharmaceuticals ltd) |
| 54472 | clarithromycin 250mg tablets (accord healthcare ltd) |
| 48163 | clarithromycin 250mg tablets (actavis uk ltd) |
| 52158 | clarithromycin 250mg tablets (alliance healthcare (distribution) ltd) |
| 54882 | clarithromycin 250mg tablets (almus pharmaceuticals ltd) |
| 52719 | clarithromycin 250mg tablets (apotex uk ltd) |
| 53086 | clarithromycin 250mg tablets (doncaster pharmaceuticals ltd) |
| 34394 | clarithromycin 250mg tablets (generics (uk) ltd) |
| 51154 | clarithromycin 250mg tablets (kent pharmaceuticals ltd) |
| 53153 | clarithromycin 250mg tablets (phoenix healthcare distribution ltd) |
| 53688 | clarithromycin 250mg tablets (ranbaxy (uk) ltd) |
| 47582 | clarithromycin 250mg tablets (sandoz ltd) |
| 50946 | clarithromycin 250mg tablets (sigma pharmaceuticals plc) |
| 54269 | clarithromycin 250mg tablets (somex pharma) |
| 34533 | clarithromycin 250mg tablets (teva uk ltd) |
| 54897 | clarithromycin 250mg tablets (tillomed laboratories ltd) |
| 53144 | clarithromycin 250mg tablets (wockhardt uk ltd) |
| 5357 | clarithromycin 250mg/5ml oral suspension |
| 54241 | clarithromycin 250mg/5ml oral suspension (a a h pharmaceuticals ltd) |
| 55148 | clarithromycin 250mg/5ml oral suspension (alliance healthcare (distribution) ltd) |
| 34811 | clarithromycin 250mg/5ml oral suspension (ranbaxy (uk) ltd) |
| 53179 | clarithromycin 250mg/5ml oral suspension (sandoz ltd) |
| 54208 | clarithromycin 250mg/5ml oral suspension (sigma pharmaceuticals plc) |
| 55428 | clarithromycin 250mg/5ml oral suspension (waymade healthcare plc) |
| 54529 | clarithromycin 500mg modified-release tablet (hillcross pharmaceuticals ltd) |
| 6803 | clarithromycin 500mg modified-release tablets |
| 681 | clarithromycin 500mg tablets |
| 38163 | clarithromycin 500mg tablets (a a h pharmaceuticals ltd) |
| 51426 | clarithromycin 500mg tablets (accord healthcare ltd) |
| 48023 | clarithromycin 500mg tablets (actavis uk ltd) |
| 49939 | clarithromycin 500mg tablets (alliance healthcare (distribution) ltd) |
| 53715 | clarithromycin 500mg tablets (almus pharmaceuticals ltd) |
| 53776 | clarithromycin 500mg tablets (doncaster pharmaceuticals ltd) |
| 34608 | clarithromycin 500mg tablets (generics (uk) ltd) |
| 53703 | clarithromycin 500mg tablets (kent pharmaceuticals ltd) |
| 46488 | clarithromycin 500mg tablets (ranbaxy (uk) ltd) |
| 40784 | clarithromycin 500mg tablets (sandoz ltd) |
| 53109 | clarithromycin 500mg tablets (somex pharma) |
| 34974 | clarithromycin 500mg tablets (teva uk ltd) |
| 53875 | clarithromycin 500mg tablets (tillomed laboratories ltd) |
| 11433 | clarithromycin 500mg with lansoprazole 30mg and amoxicillin 500mg triple pack |
| 6497 | clarithromycin 500mg with metronidazole 400mg with lansoprazole 30mg triple pack |
| 28349 | clarosip 125mg granules for oral suspension straws (grunenthal ltd) |
| 31689 | clarosip 187.5mg granules for oral suspension straws (grunenthal ltd) |
| 31690 | clarosip 250mg granules for oral suspension straws (grunenthal ltd) |
| 9925 | clavulanic acid 125mg with amoxicillin 250mg tablets |
| 13239 | clavulanic acid 125mg with amoxicillin 500mg tablets |
| 24006 | clavulanic acid 31mg with amoxcillin 125mg/5ml oral suspension |
| 21775 | clavulanic acid 31mg with amoxicillin 125mg/5ml sugar free oral suspension |
| 20432 | clavulanic acid 57mg with amoxicillin 400mg/5ml sugar free suspension |
| 42485 | clavulanic acid 62mg with amoxicillin 250mg/5ml oral suspension |
| 16612 | clavulanic acid 62mg with amoxicillin 250mg/5ml sugar free suspension |
| 24093 | clavulanic acid with amoxicillin dispersible tablets |
| 12504 | clomocycline 170mg capsules |
| 10200 | co-amoxiclav 125mg/31mg/5ml oral suspension |
| 54052 | co-amoxiclav 125mg/31mg/5ml oral suspension (a a h pharmaceuticals ltd) |
| 54732 | co-amoxiclav 125mg/31mg/5ml oral suspension (generics (uk) ltd) |
| 1638 | co-amoxiclav 125mg/31mg/5ml oral suspension sugar free |
| 43548 | co-amoxiclav 125mg/31mg/5ml oral suspension sugar free (a a h pharmaceuticals ltd) |
| 54324 | co-amoxiclav 125mg/31mg/5ml oral suspension sugar free (actavis uk ltd) |
| 54452 | co-amoxiclav 125mg/31mg/5ml oral suspension sugar free (alliance healthcare (distribution) ltd) |
| 54808 | co-amoxiclav 125mg/31mg/5ml oral suspension sugar free (almus pharmaceuticals ltd) |
| 28874 | co-amoxiclav 125mg/31mg/5ml oral suspension sugar free (ivax pharmaceuticals uk ltd) |
| 56884 | co-amoxiclav 125mg/31mg/5ml oral suspension sugar free (phoenix healthcare distribution ltd) |
| 34680 | co-amoxiclav 125mg/31mg/5ml oral suspension sugar free (ranbaxy (uk) ltd) |
| 34972 | co-amoxiclav 125mg/31mg/5ml oral suspension sugar free (sandoz ltd) |
| 829 | co-amoxiclav 250mg/125mg dispersible tablets sugar free |
| 545 | co-amoxiclav 250mg/125mg tablets |
| 30786 | co-amoxiclav 250mg/125mg tablets (a a h pharmaceuticals ltd) |
| 19209 | co-amoxiclav 250mg/125mg tablets (actavis uk ltd) |
| 51623 | co-amoxiclav 250mg/125mg tablets (alliance healthcare (distribution) ltd) |
| 48147 | co-amoxiclav 250mg/125mg tablets (almus pharmaceuticals ltd) |
| 34297 | co-amoxiclav 250mg/125mg tablets (generics (uk) ltd) |
| 28871 | co-amoxiclav 250mg/125mg tablets (ivax pharmaceuticals uk ltd) |
| 33693 | co-amoxiclav 250mg/125mg tablets (kent pharmaceuticals ltd) |
| 50446 | co-amoxiclav 250mg/125mg tablets (phoenix healthcare distribution ltd) |
| 30783 | co-amoxiclav 250mg/125mg tablets (ranbaxy (uk) ltd) |
| 19414 | co-amoxiclav 250mg/125mg tablets (sandoz ltd) |
| 34734 | co-amoxiclav 250mg/125mg tablets (teva uk ltd) |
| 55312 | co-amoxiclav 250mg/125mg tablets (waymade healthcare plc) |
| 46915 | co-amoxiclav 250mg/125mg tablets (zentiva) |
| 7364 | co-amoxiclav 250mg/62mg/5ml oral suspension |
| 54708 | co-amoxiclav 250mg/62mg/5ml oral suspension (a a h pharmaceuticals ltd) |
| 54780 | co-amoxiclav 250mg/62mg/5ml oral suspension (generics (uk) ltd) |
| 524 | co-amoxiclav 250mg/62mg/5ml oral suspension sugar free |
| 42227 | co-amoxiclav 250mg/62mg/5ml oral suspension sugar free (a a h pharmaceuticals ltd) |
| 51678 | co-amoxiclav 250mg/62mg/5ml oral suspension sugar free (almus pharmaceuticals ltd) |
| 37304 | co-amoxiclav 250mg/62mg/5ml oral suspension sugar free (ivax pharmaceuticals uk ltd) |
| 40320 | co-amoxiclav 250mg/62mg/5ml oral suspension sugar free (ranbaxy (uk) ltd) |
| 46918 | co-amoxiclav 250mg/62mg/5ml oral suspension sugar free (sandoz ltd) |
| 34234 | co-amoxiclav 250mg/62mg/5ml oral suspension sugar free (teva uk ltd) |
| 56578 | co-amoxiclav 250mg/62mg/5ml oral suspension sugar free (waymade healthcare plc) |
| 6687 | co-amoxiclav 400mg/57mg/5ml oral suspension sugar free |
| 51637 | co-amoxiclav 400mg/57mg/5ml oral suspension sugar free (a a h pharmaceuticals ltd) |
| 641 | co-amoxiclav 500mg/125mg tablets |
| 33701 | co-amoxiclav 500mg/125mg tablets (a a h pharmaceuticals ltd) |
| 50742 | co-amoxiclav 500mg/125mg tablets (actavis uk ltd) |
| 50341 | co-amoxiclav 500mg/125mg tablets (alliance healthcare (distribution) ltd) |
| 53609 | co-amoxiclav 500mg/125mg tablets (apc pharmaceuticals & chemicals (europe) ltd) |
| 53996 | co-amoxiclav 500mg/125mg tablets (aurobindo pharma ltd) |
| 30705 | co-amoxiclav 500mg/125mg tablets (generics (uk) ltd) |
| 29356 | co-amoxiclav 500mg/125mg tablets (ivax pharmaceuticals uk ltd) |
| 40148 | co-amoxiclav 500mg/125mg tablets (kent pharmaceuticals ltd) |
| 49610 | co-amoxiclav 500mg/125mg tablets (medreich plc) |
| 54591 | co-amoxiclav 500mg/125mg tablets (phoenix healthcare distribution ltd) |
| 34493 | co-amoxiclav 500mg/125mg tablets (ranbaxy (uk) ltd) |
| 32910 | co-amoxiclav 500mg/125mg tablets (sandoz ltd) |
| 29353 | co-amoxiclav 500mg/125mg tablets (teva uk ltd) |
| 44154 | co-amoxiclav 500mg/125mg tablets (zentiva) |
| 21860 | cyclodox 100mg capsule (berk pharmaceuticals ltd) |
| 21878 | demix 100 capsules (ashbourne pharmaceuticals ltd) |
| 21828 | demix 50 capsules (ashbourne pharmaceuticals ltd) |
| 2428 | distaclor 125mg/5ml liquid (dista products ltd) |
| 25384 | distaclor 125mg/5ml oral suspension (flynn pharma ltd) |
| 4576 | distaclor 250mg capsule (dista products ltd) |
| 9219 | distaclor 250mg/5ml liquid (dista products ltd) |
| 22042 | distaclor 250mg/5ml oral suspension (flynn pharma ltd) |
| 7889 | distaclor 375mg modified-release tablet (dista products ltd) |
| 319 | distaclor 500mg capsule (dista products ltd) |
| 18243 | distaclor 500mg capsules (flynn pharma ltd) |
| 3523 | distaclor 500mg modified-release tablet (dista products ltd) |
| 20992 | distaclor mr 375mg tablets (flynn pharma ltd) |
| 21038 | doxatet 100mg tablet (manufacturer unknown) |
| 2884 | doxycycline (as hyclate) 100mg dispersible tablets |
| 970 | doxycycline (as hyclate) 100mg tablets |
| 12987 | doxycycline (as hyclate) 50mg capsules with microgranules |
| 23819 | doxycycline (as hyclate) 50mg capsules with microgranules |
| 8724 | doxycycline (as hyclate) 50mg/5ml oral solution |
| 41560 | doxycycline 100mg capsule (ivax pharmaceuticals uk ltd) |
| 34594 | doxycycline 100mg capsule (neo laboratories ltd) |
| 34423 | doxycycline 100mg capsule (pliva pharma ltd) |
| 41605 | doxycycline 100mg capsule (sandoz ltd) |
| 1046 | doxycycline 100mg capsules |
| 24149 | doxycycline 100mg capsules (a a h pharmaceuticals ltd) |
| 34300 | doxycycline 100mg capsules (actavis uk ltd) |
| 49737 | doxycycline 100mg capsules (alliance healthcare (distribution) ltd) |
| 46807 | doxycycline 100mg capsules (almus pharmaceuticals ltd) |
| 32066 | doxycycline 100mg capsules (generics (uk) ltd) |
| 24126 | doxycycline 100mg capsules (ivax pharmaceuticals uk ltd) |
| 33671 | doxycycline 100mg capsules (kent pharmaceuticals ltd) |
| 53310 | doxycycline 100mg capsules (sigma pharmaceuticals plc) |
| 30739 | doxycycline 100mg capsules (teva uk ltd) |
| 55519 | doxycycline 100mg capsules (waymade healthcare plc) |
| 6396 | doxycycline 100mg dispersible tablets sugar free |
| 26747 | doxycycline 100mg tablet (neo laboratories ltd) |
| 40796 | doxycycline 40mg modified-release capsules |
| 264 | doxycycline 50mg capsules |
| 34175 | doxycycline 50mg capsules (a a h pharmaceuticals ltd) |
| 48095 | doxycycline 50mg capsules (actavis uk ltd) |
| 53973 | doxycycline 50mg capsules (alliance healthcare (distribution) ltd) |
| 34765 | doxycycline 50mg capsules (generics (uk) ltd) |
| 40391 | doxycycline 50mg capsules (ivax pharmaceuticals uk ltd) |
| 32419 | doxycycline 50mg capsules (teva uk ltd) |
| 23405 | doxylar 100mg capsules (sandoz ltd) |
| 23432 | doxylar 50mg capsules (sandoz ltd) |
| 17226 | economycin 250mg capsule (ddsa pharmaceuticals ltd) |
| 26111 | economycin 250mg tablet (ddsa pharmaceuticals ltd) |
| 40980 | efracea 40mg modified-release capsules (galderma (uk) ltd) |
| 4489 | erycen 250mg tablet (berk pharmaceuticals ltd) |
| 23017 | erycen 500mg tablet (berk pharmaceuticals ltd) |
| 318 | erymax 250mg capsule (elan pharma) |
| 10190 | erymax 250mg gastro-resistant capsules (teva uk ltd) |
| 14511 | erymax sprinkle 125mg capsule (elan pharma) |
| 9434 | erymin 250mg/5ml oral suspension (elan pharma) |
| 48017 | erythoden 125mg/5ml liquid (stevenden healthcare) |
| 41389 | erythoden 250mg/5ml liquid (stevenden healthcare) |
| 39616 | erythrocin 250 tablets (amdipharm plc) |
| 480 | erythrocin 250mg tablet (abbott laboratories ltd) |
| 1072 | erythrocin 500 500mg tablet (abbott laboratories ltd) |
| 39613 | erythrocin 500 tablets (amdipharm plc) |
| 53449 | erythrocin 500 tablets (lexon (uk) ltd) |
| 51984 | erythrocin 500 tablets (mawdsley-brooks & company ltd) |
| 53004 | erythrocin 500 tablets (necessity supplies ltd) |
| 50693 | erythrocin 500 tablets (sigma pharmaceuticals plc) |
| 50223 | erythrocin 500 tablets (stephar (u.k.) ltd) |
| 27768 | erythrolar 250mg tablet (lagap) |
| 50205 | erythrolar 250mg tablets (ennogen pharma ltd) |
| 4153 | erythrolar 250mg/5ml liquid (lagap) |
| 23954 | erythrolar 500mg tablet (lagap) |
| 49301 | erythrolar 500mg tablets (ennogen pharma ltd) |
| 3209 | erythromid 250mg tablet (abbott laboratories ltd) |
| 9148 | erythromid ds 500mg tablet (abbott laboratories ltd) |
| 1376 | erythromycin 100 mg syr |
| 7792 | erythromycin 12 mg syr |
| 14429 | erythromycin 125mg sprinkle capsules |
| 34231 | erythromycin 125mg/5ml liquid (berk pharmaceuticals ltd) |
| 33248 | erythromycin 125mg/5ml liquid (ivax pharmaceuticals uk ltd) |
| 397 | erythromycin 125mg/5ml oral suspension |
| 9656 | erythromycin 2% gel |
| 1969 | erythromycin 250 mg mix |
| 29154 | erythromycin 250mg capsule (actavis uk ltd) |
| 103 | erythromycin 250mg gastro-resistant capsules |
| 33686 | erythromycin 250mg gastro-resistant capsules (a a h pharmaceuticals ltd) |
| 50580 | erythromycin 250mg gastro-resistant capsules (actavis uk ltd) |
| 50694 | erythromycin 250mg gastro-resistant capsules (alliance healthcare (distribution) ltd) |
| 55133 | erythromycin 250mg gastro-resistant capsules (kent pharmaceuticals ltd) |
| 49952 | erythromycin 250mg gastro-resistant capsules (phoenix healthcare distribution ltd) |
| 34512 | erythromycin 250mg gastro-resistant capsules (teva uk ltd) |
| 55397 | erythromycin 250mg gastro-resistant capsules (waymade healthcare plc) |
| 34837 | erythromycin 250mg gastro-resistant tablet (co-pharma ltd) |
| 63 | erythromycin 250mg gastro-resistant tablets |
| 24127 | erythromycin 250mg gastro-resistant tablets (a a h pharmaceuticals ltd) |
| 33703 | erythromycin 250mg gastro-resistant tablets (abbott laboratories ltd) |
| 29344 | erythromycin 250mg gastro-resistant tablets (actavis uk ltd) |
| 52906 | erythromycin 250mg gastro-resistant tablets (alliance healthcare (distribution) ltd) |
| 42661 | erythromycin 250mg gastro-resistant tablets (almus pharmaceuticals ltd) |
| 52952 | erythromycin 250mg gastro-resistant tablets (co-pharma ltd) |
| 42296 | erythromycin 250mg gastro-resistant tablets (dr reddy's laboratories (uk) ltd) |
| 34334 | erythromycin 250mg gastro-resistant tablets (generics (uk) ltd) |
| 24129 | erythromycin 250mg gastro-resistant tablets (ivax pharmaceuticals uk ltd) |
| 53986 | erythromycin 250mg gastro-resistant tablets (medreich plc) |
| 55483 | erythromycin 250mg gastro-resistant tablets (milpharm ltd) |
| 52428 | erythromycin 250mg gastro-resistant tablets (phoenix healthcare distribution ltd) |
| 31530 | erythromycin 250mg gastro-resistant tablets (ranbaxy (uk) ltd) |
| 34479 | erythromycin 250mg gastro-resistant tablets (sovereign medical ltd) |
| 33685 | erythromycin 250mg gastro-resistant tablets (teva uk ltd) |
| 34873 | erythromycin 250mg tablet (berk pharmaceuticals ltd) |
| 34189 | erythromycin 250mg tablet (c p pharmaceuticals ltd) |
| 553 | erythromycin 250mg.5ml oral suspension |
| 47242 | erythromycin 250mg/5ml liquid (c p pharmaceuticals ltd) |
| 41584 | erythromycin 250mg/5ml liquid (ivax pharmaceuticals uk ltd) |
| 3408 | erythromycin 500 mg cap |
| 401 | erythromycin 500mg ec gastro-resistant tablets |
| 34869 | erythromycin 500mg tablet (c p pharmaceuticals ltd) |
| 41604 | erythromycin 500mg tablet (hillcross pharmaceuticals ltd) |
| 26365 | erythromycin 500mg tablet (ivax pharmaceuticals uk ltd) |
| 55300 | erythromycin 500mg tablet (teva uk ltd) |
| 47676 | erythromycin 500mg/5ml liquid (c p pharmaceuticals ltd) |
| 2326 | erythromycin 500mg/5ml oral suspension |
| 37796 | erythromycin estolate 125mg/5ml suspension |
| 9903 | erythromycin estolate 250mg capsules |
| 40073 | erythromycin estolate 250mg/5ml suspension |
| 37694 | erythromycin estolate 500mg tablets |
| 2429 | erythromycin ethyl succinate 125mg/5ml oral suspension |
| 13167 | erythromycin ethyl succinate 125mg/5ml oral suspension (a a h pharmaceuticals ltd) |
| 49978 | erythromycin ethyl succinate 125mg/5ml oral suspension (focus pharmaceuticals ltd) |
| 50948 | erythromycin ethyl succinate 125mg/5ml oral suspension (phoenix healthcare distribution ltd) |
| 47126 | erythromycin ethyl succinate 125mg/5ml oral suspension (pinewood healthcare) |
| 34779 | erythromycin ethyl succinate 125mg/5ml oral suspension (sandoz ltd) |
| 4672 | erythromycin ethyl succinate 125mg/5ml oral suspension sugar free |
| 33697 | erythromycin ethyl succinate 125mg/5ml oral suspension sugar free (a a h pharmaceuticals ltd) |
| 42659 | erythromycin ethyl succinate 125mg/5ml oral suspension sugar free (abbott laboratories ltd) |
| 55589 | erythromycin ethyl succinate 125mg/5ml oral suspension sugar free (alliance healthcare (distribution) ltd) |
| 48101 | erythromycin ethyl succinate 125mg/5ml oral suspension sugar free (focus pharmaceuticals ltd) |
| 33695 | erythromycin ethyl succinate 125mg/5ml oral suspension sugar free (generics (uk) ltd) |
| 34795 | erythromycin ethyl succinate 125mg/5ml oral suspension sugar free (ivax pharmaceuticals uk ltd) |
| 45870 | erythromycin ethyl succinate 125mg/5ml oral suspension sugar free (pinewood healthcare) |
| 33705 | erythromycin ethyl succinate 125mg/5ml oral suspension sugar free (teva uk ltd) |
| 2376 | erythromycin ethyl succinate 250mg/5ml oral suspension |
| 13120 | erythromycin ethyl succinate 250mg/5ml oral suspension (a a h pharmaceuticals ltd) |
| 32902 | erythromycin ethyl succinate 250mg/5ml oral suspension (kent pharmaceuticals ltd) |
| 46696 | erythromycin ethyl succinate 250mg/5ml oral suspension (sandoz ltd) |
| 2225 | erythromycin ethyl succinate 250mg/5ml oral suspension sugar free |
| 32898 | erythromycin ethyl succinate 250mg/5ml oral suspension sugar free (a a h pharmaceuticals ltd) |
| 46154 | erythromycin ethyl succinate 250mg/5ml oral suspension sugar free (abbott laboratories ltd) |
| 52860 | erythromycin ethyl succinate 250mg/5ml oral suspension sugar free (alliance healthcare (distribution) ltd) |
| 33694 | erythromycin ethyl succinate 250mg/5ml oral suspension sugar free (generics (uk) ltd) |
| 30177 | erythromycin ethyl succinate 250mg/5ml oral suspension sugar free (ivax pharmaceuticals uk ltd) |
| 34853 | erythromycin ethyl succinate 250mg/5ml oral suspension sugar free (teva uk ltd) |
| 733 | erythromycin ethyl succinate 500mg tablets |
| 2226 | erythromycin ethyl succinate 500mg/5ml oral suspension |
| 30980 | erythromycin ethyl succinate 500mg/5ml oral suspension (kent pharmaceuticals ltd) |
| 14171 | erythromycin ethyl succinate 500mg/5ml oral suspension sugar free |
| 31514 | erythromycin ethyl succinate 500mg/5ml oral suspension sugar free (abbott laboratories ltd) |
| 25595 | erythromycin ethyl succinate 500mg/5ml oral suspension sugar free (ivax pharmaceuticals uk ltd) |
| 27203 | erythromycin ethyl succinate 500mg/5ml oral suspension sugar free (teva uk ltd) |
| 25751 | erythromycin ethylsuccinate (coated) 250mg/5ml oral suspension sugar free |
| 30234 | erythromycin ethylsuccinate 125mg sachets |
| 12330 | erythromycin ethylsuccinate 1g sachets |
| 13635 | erythromycin ethylsuccinate 250mg sachets |
| 15713 | erythromycin ethylsuccinate 500mg sachets |
| 1037 | erythromycin ethylsuccinate sf 125 mg/5ml sus |
| 3907 | erythromycin sf sach 250 mg |
| 438 | erythromycin stearate 250mg tablets |
| 2350 | erythromycin stearate 500mg tablets |
| 3572 | erythroped 250mg powder (abbott laboratories ltd) |
| 16747 | erythroped 250mg sachets (abbott laboratories ltd) |
| 105 | erythroped 250mg/5ml liquid (abbott laboratories ltd) |
| 532 | erythroped 250mg/5ml oral suspension (abbott laboratories ltd) |
| 4596 | erythroped a 1g sachets (abbott laboratories ltd) |
| 327 | erythroped a 500mg tablet (abbott laboratories ltd) |
| 39632 | erythroped a 500mg tablets (amdipharm plc) |
| 54098 | erythroped a 500mg tablets (lexon (uk) ltd) |
| 56203 | erythroped a 500mg tablets (sigma pharmaceuticals plc) |
| 4372 | erythroped forte 500mg sachets (abbott laboratories ltd) |
| 993 | erythroped forte 500mg/5ml liquid (abbott laboratories ltd) |
| 4610 | erythroped forte 500mg/5ml oral suspension (abbott laboratories ltd) |
| 39642 | erythroped forte sf 500mg/5ml oral suspension (amdipharm plc) |
| 3042 | erythroped pi 125mg sachets (abbott laboratories ltd) |
| 997 | erythroped pi 125mg/5ml liquid (abbott laboratories ltd) |
| 825 | erythroped pi 125mg/5ml oral suspension (abbott laboratories ltd) |
| 39623 | erythroped pi sf 125mg/5ml oral suspension (amdipharm plc) |
| 39669 | erythroped sf 250mg/5ml oral suspension (amdipharm plc) |
| 18930 | flemoxin 375mg soluble tablet (paines & byrne ltd) |
| 24396 | flemoxin 750mg soluble tablet (paines & byrne ltd) |
| 14386 | galenamox 125mg/5ml oral suspension (galen ltd) |
| 14371 | galenamox 250mg capsules (galen ltd) |
| 14407 | galenamox 250mg/5ml oral suspension (galen ltd) |
| 14396 | galenamox 500mg capsules (galen ltd) |
| 18682 | ilosone 125mg/5ml liquid (dista products ltd) |
| 17207 | ilosone 250mg capsule (dista products ltd) |
| 19330 | ilosone 250mg/5ml liquid (dista products ltd) |
| 18643 | ilosone 500mg tablet (dista products ltd) |
| 23244 | ilotycin 250mg tablet (eli lilly and company ltd) |
| 12541 | imperacin 250mg tablet (astrazeneca uk ltd) |
| 7485 | keflex 125mg/5ml liquid (eli lilly and company ltd) |
| 27072 | keflex 125mg/5ml oral suspension (flynn pharma ltd) |
| 7430 | keflex 250mg capsule (eli lilly and company ltd) |
| 11989 | keflex 250mg capsules (flynn pharma ltd) |
| 9157 | keflex 250mg tablet (eli lilly and company ltd) |
| 830 | keflex 250mg tablets (flynn pharma ltd) |
| 10455 | keflex 250mg/5ml liquid (eli lilly and company ltd) |
| 28722 | keflex 250mg/5ml oral suspension (flynn pharma ltd) |
| 12276 | keflex 500mg capsule (eli lilly and company ltd) |
| 24618 | keflex 500mg capsules (flynn pharma ltd) |
| 9603 | keflex 500mg tablet (eli lilly and company ltd) |
| 31110 | keflex 500mg tablets (flynn pharma ltd) |
| 26233 | keftid 125mg/5ml oral suspension (co-pharma ltd) |
| 26207 | keftid 250mg capsules (co-pharma ltd) |
| 41853 | keftid 250mg/5ml oral suspension (co-pharma ltd) |
| 26236 | keftid 500mg capsules (co-pharma ltd) |
| 33304 | kerymax 250mg gastro-resistant capsules (kent pharmaceuticals ltd) |
| 26989 | kiflone 125mg/5ml oral solution (berk pharmaceuticals ltd) |
| 21835 | kiflone 250mg capsule (berk pharmaceuticals ltd) |
| 21979 | kiflone 250mg/5ml oral solution (berk pharmaceuticals ltd) |
| 27017 | kiflone 500mg capsule (berk pharmaceuticals ltd) |
| 26992 | kiflone 500mg tablet (berk pharmaceuticals ltd) |
| 3736 | klaricid 125mg/5ml oral suspension (abbott laboratories ltd) |
| 2719 | klaricid 250mg tablets (abbott laboratories ltd) |
| 52411 | klaricid 250mg tablets (necessity supplies ltd) |
| 9583 | klaricid 250mg/5ml oral suspension (abbott laboratories ltd) |
| 6623 | klaricid 500 tablets (abbott laboratories ltd) |
| 14816 | klaricid adult 250mg granules sachets (abbott laboratories ltd) |
| 38997 | klaricid paediatric 125mg/5ml oral suspension (abbott laboratories ltd) |
| 39010 | klaricid paediatric 250mg/5ml oral suspension (abbott laboratories ltd) |
| 6121 | klaricid xl 500mg tablets (abbott laboratories ltd) |
| 15290 | lansoprazole with amoxicillin and clarithromycin 30mg + 500mg + 500mg triple pack |
| 7439 | ledermycin 150mg capsule (wyeth pharmaceuticals) |
| 16613 | ledermycin 150mg capsules (mercury pharma group ltd) |
| 22076 | ledermycin 300mg tablet (wyeth pharmaceuticals) |
| 6295 | levofloxacin 250mg tablets |
| 55708 | levofloxacin 250mg tablets (actavis uk ltd) |
| 56012 | levofloxacin 250mg tablets (dr reddy's laboratories (uk) ltd) |
| 5238 | levofloxacin 500mg tablets |
| 53673 | levofloxacin 500mg/100ml infusion bags |
| 19001 | megaclor 170mg capsule (pharmax ltd) |
| 6306 | moxifloxacin 400mg tablets |
| 17222 | mysteclin oral solution (bristol-myers squibb pharmaceuticals ltd) |
| 15071 | nordox 100mg capsule (sankyo pharma uk ltd) |
| 8393 | novobiocin/tetracycline 125 mg cap |
| 25752 | nystatin with tetracycline hc capsule |
| 9361 | oxymycin 250mg tablets (dr reddy's laboratories (uk) ltd) |
| 2458 | oxytetracycline 100 mg tab |
| 9034 | oxytetracycline 125mg/5ml syrup |
| 8285 | oxytetracycline 250 mg syr |
| 132 | oxytetracycline 250mg capsules |
| 34888 | oxytetracycline 250mg tablet (c p pharmaceuticals ltd) |
| 77 | oxytetracycline 250mg tablets |
| 34044 | oxytetracycline 250mg tablets (a a h pharmaceuticals ltd) |
| 34040 | oxytetracycline 250mg tablets (actavis uk ltd) |
| 34336 | oxytetracycline 250mg tablets (ivax pharmaceuticals uk ltd) |
| 40483 | oxytetracycline 250mg tablets (sandoz ltd) |
| 34141 | oxytetracycline 250mg tablets (teva uk ltd) |
| 28291 | oxytetracycline 3%/hydrocortisone 1% |
| 10542 | oxytetracycline hcl/hydrocortisone .5 % ear |
| 17703 | oxytetramix 250 tablets (ashbourne pharmaceuticals ltd) |
| 30520 | primacine 125mg/5ml liquid (pinewood healthcare) |
| 39118 | primacine 250mg/5ml liquid (pinewood healthcare) |
| 27504 | primacine 500mg/5ml liquid (pinewood healthcare) |
| 27681 | ranclav 125mg/31mg/5ml sf oral suspension (ranbaxy (uk) ltd) |
| 25370 | ranclav 375mg tablets (ranbaxy (uk) ltd) |
| 22017 | respillin 125mg/5ml oral solution (opd pharm) |
| 22015 | respillin 125mg/5ml oral solution (opd pharm) |
| 24203 | respillin 250mg capsule (opd pharm) |
| 24200 | respillin 500mg capsule (opd pharm) |
| 31428 | retcin 250mg tablet (ddsa pharmaceuticals ltd) |
| 21808 | rommix 125mg/5ml oral suspension sugar free (ashbourne pharmaceuticals ltd) |
| 11611 | rommix 250 ec tablets (ashbourne pharmaceuticals ltd) |
| 25278 | rommix 500mg tablet (ashbourne pharmaceuticals ltd) |
| 24097 | rondomycin 150mg capsule (pfizer ltd) |
| 18109 | sebomin mr 100mg capsules (actavis uk ltd) |
| 37440 | sebren mr 100mg capsules (teva uk ltd) |
| 19693 | sustamycin 250mg capsule (boehringer mannheim uk ltd) |
| 17693 | tavanic 250mg tablets (sanofi) |
| 6206 | tavanic 500mg tablets (sanofi) |
| 27254 | tenkorex 500mg capsule (opd pharm) |
| 7455 | terramycin 250mg capsule (pfizer ltd) |
| 17467 | terramycin 250mg tablets (pfizer ltd) |
| 9014 | tetrabid-organon 250mg capsule (organon laboratories ltd) |
| 8219 | tetrachel 250mg capsule (berk pharmaceuticals ltd) |
| 3816 | tetrachel 250mg tablet (berk pharmaceuticals ltd) |
| 25017 | tetracycline |
| 56044 | tetracycline 125mg/5ml oral solution |
| 8284 | tetracycline 125mg/5ml syrup |
| 21804 | tetracycline 125mg/5ml syrup |
| 41547 | tetracycline 250mg capsule (berk pharmaceuticals ltd) |
| 121 | tetracycline 250mg capsules |
| 34011 | tetracycline 250mg capsules |
| 56181 | tetracycline 250mg tablet (celltech pharma europe ltd) |
| 45271 | tetracycline 250mg tablet (numark management ltd) |
| 386 | tetracycline 250mg tablets |
| 43538 | tetracycline 250mg tablets (a a h pharmaceuticals ltd) |
| 41636 | tetracycline 250mg tablets (actavis uk ltd) |
| 54214 | tetracycline 250mg tablets (alliance healthcare (distribution) ltd) |
| 53117 | tetracycline 250mg tablets (almus pharmaceuticals ltd) |
| 48100 | tetracycline 250mg tablets (teva uk ltd) |
| 2922 | tetracycline 250mg with nystatin 250000units tablets |
| 2636 | tetracycline 500 mg cap |
| 3528 | tetracycline 500 mg tab |
| 21654 | tetracycline ear/eye |
| 21629 | tetracycline eye |
| 31425 | tetracycline hcl/pancreatic concentrate cap |
| 28736 | tetracycline hydrochloride/amphotericin syr |
| 15355 | tetracycline with chlortetracycline & demeclocycline tablets |
| 25071 | tetracycline with nystatin capsules |
| 4951 | tetralysal 300 capsules (galderma (uk) ltd) |
| 20054 | tetralysal 408mg capsule (pharmacia ltd) |
| 25280 | tiloryth 250mg gastro-resistant capsules (tillomed laboratories ltd) |
| 268 | vibramycin 100mg capsules (pfizer ltd) |
| 3152 | vibramycin 100mg dispersible tablet (pfizer ltd) |
| 10454 | vibramycin 50mg/5ml oral solution (pfizer ltd) |
| 9267 | vibramycin acne pack 50mg capsules (pfizer ltd) |
| 56198 | vibramycin-d 100mg dispersible tablets (mawdsley-brooks & company ltd) |
| 14904 | vibramycin-d 100mg dispersible tablets (pfizer ltd) |
| 52967 | vibramycin-d 100mg dispersible tablets (stephar (u.k.) ltd) |
| 53135 | vibramycin-d 100mg dispersible tablets (waymade healthcare plc) |
| 26392 | vibrox 100mg capsules (kent pharmaceuticals ltd) |
| 21829 | zoxycil 250mg capsule (trinity pharmaceuticals ltd) |
| 26262 | zoxycil 500mg capsule (trinity pharmaceuticals ltd) |

**CODES USED TO IDENTIFY ANNUAL REVIEWS AND RESCUE PACK PRESCRIPTIONS**

| **Medical code** | **Read term** |
| --- | --- |
| 9520 | Chronic obstructive pulmonary disease monitoring |
| 10043 | Asthma annual review |
| 11287 | Chronic obstructive pulmonary disease annual review |
| 25997 | Deferred antibiotic therapy |
| 28743 | Number of COPD exacerbations in past year |
| 100459 | Advance supply of steroid medication |
| 101042 | Issue of chronic obstructive pulmonary disease rescue pack |
